# Toxicity of fluoride: critical evaluation of evidence for human developmental neurotoxicity in epidemiological studies, animal experiments and in vitro analyses

**DOI:** 10.1007/s00204-020-02725-2

**Published:** 2020-05-08

**Authors:** Sabine Guth, Stephanie Hüser, Angelika Roth, Gisela Degen, Patrick Diel, Karolina Edlund, Gerhard Eisenbrand, Karl-Heinz Engel, Bernd Epe, Tilman Grune, Volker Heinz, Thomas Henle, Hans-Ulrich Humpf, Henry Jäger, Hans-Georg Joost, Sabine E. Kulling, Alfonso Lampen, Angela Mally, Rosemarie Marchan, Doris Marko, Eva Mühle, Michael A. Nitsche, Elke Röhrdanz, Richard Stadler, Christoph van Thriel, Stefan Vieths, Rudi F. Vogel, Edmund Wascher, Carsten Watzl, Ute Nöthlings, Jan G. Hengstler

**Affiliations:** 1 Department of Toxicology, Leibniz Research Centre for Working Environment and Human Factors (IfADo), Dortmund, Germany; 2 Department of Molecular and Cellular Sports Medicine, Institute of Cardiovascular Research and Sports Medicine, German Sport University Cologne, Cologne, Germany; 3 Kühler Grund 48/1, 69126 Heidelberg, Germany; 4 Department of General Food Technology, School of Life Sciences, TU Munich, Freising, Germany; 5 Institute of Pharmacy and Biochemistry, University of Mainz, Mainz, Germany; 6 Department of Molecular Toxicology, German Institute of Human Nutrition (DIfE), Nuthetal, Germany; 7 German Institute of Food Technologies (DIL), Quakenbrück, Germany; 8 Department of Food Chemistry, TU Dresden, Dresden, Germany; 9 Institute of Food Chemistry, Westfälische Wilhelms-Universität Münster, Münster, Germany; 10 Institute of Food Technology, University of Natural Resources and Life Sciences (BOKU), Vienna, Austria; 11 Department of Experimental Diabetology, German Institute of Human Nutrition (DIfE), Nuthetal, Germany; 12 Department of Safety and Quality of Fruit and Vegetables, Max Rubner-Institut, Federal Research Institute of Nutrition and Food, Karlsruhe, Germany; 13 Department of Food Safety, Bundesinstitut für Risikobewertung (BfR), Berlin, Germany; 14 Department of Toxicology, University of Würzburg, Würzburg, Germany; 15 Department of Food Chemistry and Toxicology, Faculty of Chemistry, University of Vienna, Vienna, Austria; 16 Department of Psychology and Neurosciences, Leibniz Research Centre for Working Environment and Human Factors (IfADo), Dortmund, Germany; 17 Department of Neurology, University Medical Hospital Bergmannsheil, Ruhr-University, Bochum, Germany; 18 Department of Experimental Pharmacology and Toxicology, Federal Institute for Drugs and Medical Devices (BfArM), Bonn, Germany; 19 Institute of Food Safety and Analytic Sciences, Nestlé Research Centre, Lausanne, Switzerland; 20 Paul-Ehrlich-Institut, Langen, Germany; 21 Lehrstuhl für Technische Mikrobiologie, TU Munich, Freising, Germany; 22 Department of Ergonomics, Leibniz Research Centre for Working Environment and Human Factors (IfADo), Dortmund, Germany; 23 Department of Immunology, Leibniz Research Centre for Working Environment and Human Factors (IfADo), Dortmund, Germany; 24 Department of Nutrition and Food Sciences, Nutritional Epidemiology, Rheinische Friedrich-Wilhelms University Bonn, Bonn, Germany

**Keywords:** Sodium fluoride, Developmental neurotoxicity, Epidemiological studies, Animal studies, In vitro data, Risk assessment

## Abstract

Recently, epidemiological studies have suggested that fluoride is a human developmental neurotoxicant that reduces measures of intelligence in children, placing it into the same category as toxic metals (lead, methylmercury, arsenic) and polychlorinated biphenyls. If true, this assessment would be highly relevant considering the widespread fluoridation of drinking water and the worldwide use of fluoride in oral hygiene products such as toothpaste. To gain a deeper understanding of these assertions, we reviewed the levels of human exposure, as well as results from animal experiments, particularly focusing on developmental toxicity, and the molecular mechanisms by which fluoride can cause adverse effects. Moreover, in vitro studies investigating fluoride in neuronal cells and precursor/stem cells were analyzed, and 23 epidemiological studies published since 2012 were considered. The results show that the margin of exposure (MoE) between no observed adverse effect levels (NOAELs) in animal studies and the current adequate intake (AI) of fluoride (50 µg/kg b.w./day) in humans ranges between 50 and 210, depending on the specific animal experiment used as reference. Even for unusually high fluoride exposure levels, an MoE of at least ten was obtained. Furthermore, concentrations of fluoride in human plasma are much lower than fluoride concentrations, causing effects in cell cultures. In contrast, 21 of 23 recent epidemiological studies report an association between high fluoride exposure and reduced intelligence. The discrepancy between experimental and epidemiological evidence may be reconciled with deficiencies inherent in most of these epidemiological studies on a putative association between fluoride and intelligence, especially with respect to adequate consideration of potential confounding factors, e.g., socioeconomic status, residence, breast feeding, low birth weight, maternal intelligence, and exposure to other neurotoxic chemicals. In conclusion, based on the totality of currently available scientific evidence, the present review does not support the presumption that fluoride should be assessed as a human developmental neurotoxicant at the current exposure levels in Europe.

**Electronic supplementary material:**

The online version of this article (10.1007/s00204-020-02725-2) contains supplementary material, which is available to authorized users.

## Introduction

Since the 1940s, fluoride has been added to drinking water in many countries to reduce dental caries. Since then, the benefits and risks of fluoride remain among the most frequently discussed topics in the field of public health. This high interest is illustrated by the number of articles retrieved from a PubMed search (January 2019), which yielded 1416 articles for the keywords ‘fluoride AND toxicity’ since 2000, and 472 articles published since 2015. In recent years, the possible adverse health effects of fluoride have gained attention as indicated by the increased number of scientific publications and reports from different media outlets, some of which highly recommend to not ‘take up any fluoride, particularly not during pregnancy’. Some extreme examples, but also examples of balanced and objective reports, are documented in Online Resource 1. Frequently included in many reports is the statement that one of the world’s leading medical journals now ‘officially assessed fluoride as a human developmental neurotoxicant’. In this context, an article published in *Lancet Neurology* is often used as a reference, in which the authors claim that since 2006, epidemiological studies have documented additional human developmental neurotoxicants, among them fluoride, which apparently should now be placed in the same category as toxic metals (lead, methylmercury, arsenic) and polychlorinated biphenyls (Grandjean and Landrigan 2014). Moreover, further epidemiological publications—usually with a cross-sectional study design—report an association between high exposure to fluoride via drinking water and low intelligence. In the present article, we reviewed the available literature to critically evaluate the human health hazards caused by exposure to fluoride, particularly focusing on developmental toxicity. Epidemiological studies, animal experiments and in vitro studies were considered to provide this comprehensive assessment.

## Toxicity of fluoride: the basics

### Occurrence

Fluoride (F^−^) is an inorganic anion that naturally occurs in minerals, particularly in fluorite (CaF_2_). Fluoride salts are highly soluble and found ubiquitously in water, varying widely in concentration. For example, the levels in surface water are usually below 0.5 mg/L, while much wider ranges (0.1 and 6 mg/L) have been reported in groundwater (EFSA 2013). Depending on the presence of certain minerals, concentrations greater than 10 mg/L have been observed; however, such high concentrations are rare. Seawater also contains fluoride, but within a relatively narrow range between 1.2 and 1.5 mg/L (EFSA 2013).

### Absorption, excretion, and accumulation

Soluble fluorides, e.g., sodium fluoride (NaF), are almost completely absorbed from the gastrointestinal tract into the blood (Barbier et al. 2010; EFSA 2005), with peak plasma levels attained within 20–60 min after oral ingestion (EFSA 2005; Whitford et al. 2008). Uptake may however be reduced by the formation of insoluble complexes or precipitates with food components. The presence of calcium in milk, for example, reduces systemic absorption. Fluoride is able to cross biological membranes by diffusion as the non-ionic hydrogen fluoride (HF) (Gutknecht and Walter 1981). The pKa of HF is approximately 3.4; therefore, more of the non-ionic HF is present in acidic rather than in alkaline compartments (Buzalaf and Whitford 2011; Whitford 1996). The largest amount of absorbed fluoride is retained in bone and teeth (ATSDR 2003), where about 99% of the total fluoride in an organism are found (Ekstrand et al. 1977). In rats, the ratio of fluoride in soft tissues to plasma ranges between 0.4 and 0.9 (Whitford et al. 1979); reviewed by EFSA (EFSA 2013). However, the blood-brain barrier has a relatively low permeability, leading to ratios of approximately 0.1 between brain tissue and plasma. In contrast, the kidney may contain higher fluoride concentrations compared to plasma (Taves et al. 1983). Fluoride has also been reported to cross the placenta, and early reports have indicated that supplements of 1.5 mg fluoride/day may increase fetal blood concentrations approximately twofold (Caldera et al. 1988; Shen and Taves 1974). Finally, most of the absorbed fluoride is excreted by the kidney, and only a smaller fraction via the feces (Villa et al. 2010).

### Mechanisms of action

Fluoride interacts with proteins, particularly enzymes, and usually inhibits enzyme activity at concentrations in the millimolar range (Barbier et al. 2010; Mendoza-Schulz et al. 2009). However, cell proliferation may be stimulated at concentrations in the micromolar range (Adamek et al. 2005; Mendoza-Schulz et al. 2009). Whether fluoride has an essential function in cells or organisms is not known. The mechanisms by which fluoride affects cell functions include the generation of superoxide anions (Garcia-Montalvo et al. 2009; Izquierdo-Vega et al. 2008); mitochondrial toxicity, e.g., opening of the transition pore (Anuradha et al. 2001); release of cytochrome c from mitochondria and induction of apoptosis (Chlubek et al. 2003; Lee et al. 2008); inhibition of migration, e.g., of embryonic neurons (Horgan et al. 1994) and sperm (Izquierdo-Vega et al. 2008); increased endoplasmic reticulum stress in ameloblasts, the cell type responsible for enamel formation (Kubota et al. 2005); increased expression of inflammatory factors, such as NF-kappaB (Zhang et al. 2008) and IL-8 (Schwarze et al. 2000); and the modified release of the neurotransmitters acetylcholine (Flora et al. 2009) and gamma-aminobutyric acid (Gardiner and de Belleroche 1990).

At high doses, NaF has been shown to affect the immune system in mice (Guo et al. 2017). Doses higher than 12 mg/kg NaF resulted in a significant decrease in the percentages of T and B lymphocytes in peripheral blood. Moreover, a decrease in the serum concentration of the cytokines interleukin (IL)-2, IL-4, IL-6, IL-10, IL-17A, interferon (IFN)-γ, and tumor necrosis factor (TNF) was observed (Guo et al. 2017). In line with the reduction of B lymphocytes, NaF caused a decrease of antibody (IgA, IgG and IgM) concentrations in serum (Guo et al. 2017).

**Fig. 1:**
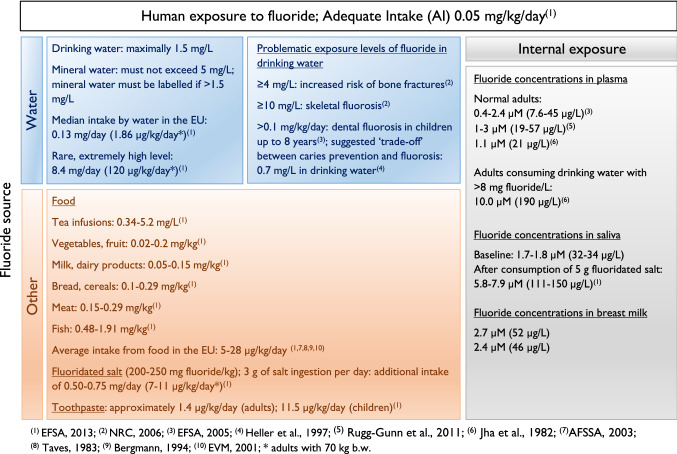
Human exposure to fluoride

Specific molecular targets for most of the effects of fluoride remain to be established and many of the findings from in vitro studies were only observed in the millimolar range. Examples include studies with human pulmonary epithelial cells, human hepatocellular carcinoma cells, rat hippocampal neurons, and mouse hepatocytes, where fluoride-induced effects (e.g., induction of cyclooxygenase 2, p53, heat shock protein 70, NF-kappaB, decrease of glutathione) were observed at 2.1, 3, 5, and 100 mM, as reviewed by Barbier et al. (Barbier et al. 2010). The in vivo relevance of such concentrations in humans is questionable, since fluoride plasma concentrations in healthy adults generally range between 0.4 and 3.0 µM and it is likely that the soft tissue concentration is even lower (Fig. 1). Furthermore, even in patients with dental and skeletal fluorosis (see below), concentrations usually do not increase more than 20-fold above these reference levels (EFSA 2005). However, some of the effects on dental and skeletal cells identified in vitro were obtained using close to in vivo relevant concentrations. Examples include increased proliferation of ameloblasts (Yan et al. 2007), which was observed at micromolar fluoride concentrations, decreased expression of matrix metalloproteinase-20 in human ameloblasts (10 µM), and increased expression of osteoclast differentiation factor in cultivated rat osteoblasts (50 µM), as reviewed by Barbier et al. (Barbier et al. 2010).

### Positive health effects

Currently, there is no known essential function for fluoride in cells and organisms. However, experimental studies to determine whether fluoride is indispensable are challenging, because it is difficult to completely avoid fluoride uptake. In one study, rats (F344) were kept under low-fluoride conditions (2–23 µg/kg b.w./day) for several generations, which led to decreased weight gain (Schwarz and Milne 1972). In another study, supplementing fluoride to breastfed children in an area with low fluoride in the drinking water and breast milk was reported to significantly increase the height and weight of the children (Bergmann 1994). The preventive effect of fluoride against caries at 0.05 mg/kg b.w./day will be discussed below. Nevertheless, none of these observations proves an essential role for fluoride.

## Human exposure

### Water

The main source of human exposure to fluoride is water (EFSA 2013). The median fluoride intake via water and water-based beverages in EU countries is 0.13 mg/day, which corresponds to 1.86 µg/kg b.w./day for an adult weighing 70 kg (Fig. 1). An extreme case is the intake of approximately 8.4 mg/day (120 µg/kg b.w./day for a person of 70 kg), which was calculated based on the highest observed consumption (97.5th percentile) of tap water and a fluoride concentration of 3 mg/L (EFSA 2013; SCHER 2011). According to the German drinking water ordinance, fluoride concentrations in tap water must not exceed 1.5 mg fluoride/L. This value is only rarely exceeded and most samples of drinking water are below 0.3 mg fluoride/L (BMG/UBA 2015; Schleyer and Kerndorf 1992). According to the European Commission Directive 2003/40/EC, the fluoride content of natural mineral water must not exceed 5 mg/L, and it must be labeled if it contains more than 1.5 mg/L. In Europe, fluoride is only added to drinking water in some regions of the UK, Spain, Portugal and Ireland. In the USA, the Environmental Protection Agency (EPA) recommends a concentration of 0.7 mg/L (PHS 2015).

### Food

Food generally contains low fluoride concentrations in the range of 0.1–0.5 mg/kg (EFSA 2013). Typical amounts of fluoride in foods are depicted in Fig. 1. The fluoride content of both fish and meat depends on the care taken with deboning and can be as high as 5 mg/kg (EFSA 2013). Dried herbs, which are eaten in small amounts only, contain up to 2.0 mg fluoride/kg (EFSA 2013). Black and green tea may contain 170–400 mg fluoride per kg dry weight, with concentrations in tea infusions ranging between 0.34 and 5.2 mg/L (Chan and Koh 1996; Schmidt and Funke 1984). Finally, fluoridated salt contains 200–250 mg fluoride per kg, and on its own may contribute an additional fluoride intake of approximately 0.5–0.75 mg/day (7–11 µg/kg b.w./day for a person of 70 kg) (EFSA 2013). The consumption of fluoridated salt differs between countries. In Switzerland and Germany, ~ 85% and ~ 67% of the domestic salt is fluoridated, whereas fluoridated salt is only rarely used in other European countries (Marthaler 2013). In Latin America, more than 100 million users of fluoridated salt were reported and in several countries around 90–99% coverage was achieved (Marthaler 2013).

### Exposure from dietary sources in Europe

Reliable and representative data on the total fluoride intake of the European population are not available (EFSA 2013). In France, the intake of fluoride through food (water, toothpaste, and supplements excluded) was estimated to be about 2 mg/day for adults (29 µg/kg b.w./day for a person of 70 kg) (AFSSA 2003). In the UK, the average total dietary fluoride intake of the adult population, including tea but excluding drinking water, was estimated from the 1997 Total Diet Study to be 1.2 mg/day (17 µg/kg b.w./day for a person of 70 kg) (EFSA 2013; EVM 2001). Earlier, a fluoride intake of 1.78 mg/day from both food and beverages (25 µg/kg b.w. for a person of 70 kg) and of 0.4 mg/day from foods only (6 µg/kg b.w./day for a person of 70 kg) for adults in the UK had been estimated (EFSA 2013; Taves 1983). In Sweden, the fluoride intake of adults from food and beverages in areas with low fluoride concentrations in drinking water (< 0.4 mg/L) was estimated to be 0.4–1.0 mg/day (6–14 µg/kg b.w./day for a person of 70 kg), while in areas with fluoride concentrations of 1 mg/L in the water the mean intake was estimated to be 2.1–4.4 mg/day (30–63 µg/kg b.w./day for a person of 70 kg) (Becker and Bruce 1981; EFSA 2013). In Germany, the dietary fluoride intake (solids and beverages) was estimated to be 0.379 mg/day in adults (5 µg/kg b.w./day for a person of 70 kg) (Bergmann 1994; EFSA 2013). This intake was increased considerably when a high fluoride concentration of 1 mg/L in drinking water was present or fluoridated salt was used (0.25 mg fluoride per gram of salt) (Bergmann 1994; EFSA 2013).

Recently, a total diet study on fluoride intake in Ireland that considered exposure from foods, beverages, and fluoridated water was carried out among children aged 1–12 years, as well as in adults (FSAI 2018). Mean fluoride exposures among preschool children (1–4 years of age) and children (5–12 years of age) were 23 and 17 µg/kg b.w./day, respectively, which were lower than levels measured in adults (40 µg/kg b.w./day). The higher exposure of adults was predominantly due to fluoride consumption by black tea that contributed approximately 76% of the total exposure.

Overall, the average exposure in European areas with low fluoride in drinking water was estimated to be in the range of 5–14 µg/kg b.w./day, whereas in areas with high fluoride in drinking water an average exposure of approximately 30–40 µg/kg b.w./day (maximum: 63 µg/kg b.w./day) was estimated. Therefore, the mean intake of fluoride from food, water, and beverages generally was below the adequate intake (AI) level of 50 µg/kg b.w., which is recommended for caries protection for all age groups, and particularly for children. This level could slightly be exceeded in areas with high fluoride in drinking water (≥ 1 mg/L) and maximum intake levels.

### Oral hygiene products

Fluoride-containing toothpaste, gels, and rinses may increase total fluoride intake. Small children and some adults tend to swallow toothpaste, which has been estimated to add between 0.016 and 0.15 mg fluoride uptake per cleaning procedure (EFSA 2013). Toothpaste can account for up to 25% of the total systemic dose for children aged between 2 and 6 years, depending on the amount of toothpaste swallowed during brushing (SCHER 2011). The average intake of fluoride from toothpaste was estimated to be approximately 1.4 µg/kg b.w./day for adults and 11.5 µg/kg b.w./day for children (EFSA 2013).

### Biomarkers of body burden

Fluoride concentrations in plasma are influenced by the current intake, with 0.4–3.0 µM being reported (IPCS 2002; Rugg-Gunn et al. 2011; Whitford 1996) (Fig. 1). Concentrations of fluoride in human plasma increase with the fluoride content in bone, with age, and as a consequence of renal insufficiency (Ekstrand and Whitford 1988). They may be up to 20-fold higher in individuals with skeletal and dental fluorosis (Jha et al. 1982). In breast milk, concentrations of 2.7 µM (52 µg/L) and 2.4 µM (46 µg/L) have been reported in fluoridated and non-fluoridated areas, respectively (Dirks et al. 1974; Ekstrand et al. 1981; Koparal et al. 2000). Baseline concentrations of fluoride in saliva have been reported to be 1.7–1.8 µM (EFSA 2013), which may increase to 5.8–7.9 µM after consumption of approximately 5 g of fluoridated salt (0.25 mg fluoride per gram of salt).

## Human toxicity

### Acute toxicity

Symptoms due to toxicity include respiratory arrest, cardiac depression, vomiting, diarrhea, and salivation. In humans, lethal doses have been reported in the range of 40–80 mg/kg b.w. (Boink et al. 1994; Eichler et al. 1982; Lidbeck et al. 1943; Simpson et al. 1980; Whitford 1996). This knowledge stems from mass poisoning catastrophes. For example, in 1943, 163 prison inmates were accidentally poisoned resulting in 47 fatalities. In this unfortunate incident, eggs were accidentally prepared with cockroach powder containing sodium fluoride rather than the usual milk powder. Non-lethal overdosing has also been observed in the range of 0.4–5 mg/kg b.w. (for example, by accidental overdosing of caries prophylaxis tablets), and has been reported to cause nausea and gastrointestinal effects (Eichler et al. 1982; Whitford 1996).

### Dental and skeletal fluorosis

One of the best documented long-term effects of fluoride in humans is dental fluorosis (EFSA 2005, 2013). Excessive fluoride incorporation into dental enamel before the eruption of teeth leads to hypomineralization of the developing teeth. Susceptibility to dental fluorosis ends at about 8 years of age when enamel maturation is completed. The risk of dental fluorosis should be evaluated in relation to the caries preventive effect of fluoride. Knowledge in this field stems from studies completed before 1980, when endemic fluoride in drinking water was the only relevant source of human fluoride intake (EFSA 2013). These studies demonstrated that the prevalence of caries was negatively correlated with the fluoride concentration in drinking water, with a maximal preventive effect at 1 mg/L. At this fluoride concentration in drinking water, 10% of the study population exhibited mild dental fluorosis (EFSA 2013). Balancing the benefits of caries prevention against the risk of dental fluorosis, EFSA recommended an AI of 0.05 mg fluoride/kg b.w. per day from all sources for children and adults, including pregnant and lactating women (EFSA 2013). For adults, this fluoride intake is not exceeded with a drinking water concentration of approximately 1 mg/L fluoride, under conditions where drinking water is the only relevant source of fluoride. For children, however, the AI may just be reached, for example when a 6-year-old child weighing 20 kg drinks 1 L of water containing 1 mg fluoride/L.

Skeletal fluorosis is a reversible effect characterized by deficient mineralization of the bone, leading to changes in bone structure and increased risk of fractures. Skeletal fluorosis is endemic in several countries where the potable water sources naturally contain high fluoride levels (> 4 mg/L), and where water consumption is high due to hot climates (EFSA 2013). Fluoride intakes of above 6–8 mg/day may increase the risk of bone fractures (EFSA 2013; NHMRC 2017a; WHO 2011, 2017).

### Carcinogenicity

A series of epidemiological studies addressed the question whether high fluoride in drinking water is associated with cancer mortality, but none reported a significant association (IARC 1982; Knox 1985). Studies on fluoride conducted in vitro and in vivo have reported some evidence of genotoxicity, but no causal link between high fluoride intake and increased human cancer risk was ever established (EFSA 2008).

### Reproductive and developmental toxicity

Extremely high exposure to 38.5 mg fluoride/L in drinking water was reported to be associated with infertility in men (Neelam et al. 1987). Furthermore, in recent years a relatively large number of studies have been published—as discussed in the next section—which reports that high fluoride intake is associated with reduced IQ in children.

## Studies with experimental animals

**Fig. 2:**
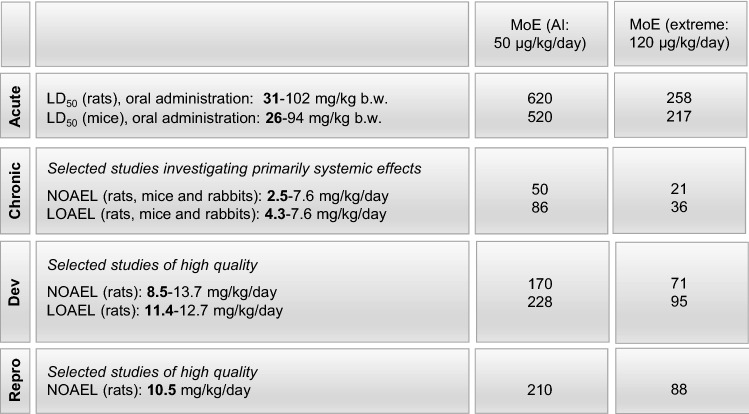
Overview of animal studies with fluoride (F^−^) with regard to acute, chronic, developmental (Dev) and reproductive (Repro) toxicity

To accurately interpret evidence of fluoride toxicity in humans obtained from epidemiological studies, we sought to compare known human exposure levels to NOAELs and LOAELs derived from experimental animal studies. Therefore, we reviewed animal studies that included acute and chronic toxicity data, as well as data on developmental, neurobehavioral, and reproductive toxicity (Fig. 2; Tables 1, 2, 3, 4, 5). The LD_50_ (lethal dose 50%) of sodium fluoride after oral administration ranges between 31 and 102 mg/kg b.w./day in rats (ATSDR 1993; IARC 1982), and between 26 and 94 mg/kg b.w./day in mice (IARC 1982; Whitford 1990). Thus, acute toxicity (LD_50_) occurred between 26 and 102 mg/kg b.w./day, and chronic toxicity (LOAEL) between 4.3 and 7.6 mg/kg b.w./day fluoride (Fig. 2) (NRC 2006). Developmental toxicity from four comprehensive studies—selected because of their compliance to standard guidelines and the use of adequate numbers of animals (NRC 2006)—was found to be in a range between 11.4 and 12.7 mg/kg b.w./day (LOAELs). In the following paragraphs, overviews of the available chronic, developmental, neurobehavioral, and reproductive toxicity studies are provided.

**Table 1:** Chronic toxicity studies; selected studies investigating primarily systemic effects

Species, strain, number of animals	Exposure duration, chemical form, route	NaF concentration in DW (mg/L)	NaF doses^a^ (mg/kg b.w./day)	Effects	NOAEL F^–^ (mg/kg b.w./day)	LOAEL F^–^ (mg/kg b.w./day)	Comment	References
Rat F-344 N 50–80 sex/group	103 weeks Sodium fluoride (DW)	0, 25, 100, 175 (0, 11.3, 45.3, 79.3 mg F^–^/L) Control: deionized water	0, 1.3, 5.2–5.5, 8.6–9.5 (corresponding to 0, 0.6, 2.4–2.5, 3.9–4.3 mg F^–^/kg b.w./day)	Resp, cardio, gastro, hemato, hepatic, renal, bd wt	3.9		Diet: NIH-07 low fluoride	NTP (1990)
				Musc/skel (osteosclerosis)	2.5	4.3		
Mouse B6C3F1 50–80 sex/group	103 weeks Sodium fluoride (DW)	0, 25, 100, 175 (0, 11.3, 45.3, 79.3 mg F^–^/L) Control: deionized water	0, 2.4–2.8, 9.6–11.3, 16.7–18.8 (corresponding to 0,1.1–1.3, 4.3–5.1, 7.6–8.5 mg F^–^/kg b.w./day)	Resp, cardio, gastro, hemato, hepatic, renal, bd wt	7.6		Diet: NIH-07 low fluoride	NTP (1990)
				Musc/skel (dentine dysplasia)	4.3, M	7.6		
Rabbit 9/group	24 month Sodium fluoride (GW)		10 (corresponding to 4.5 mg F^–^/kg b.w./day)	Gastro (roughened duodena mucosa)		4.5	Standard animal diet; DW: fluoride content less than 0.5 mg/L	Susheela and Das (1988)
Rabbit 5/group	7–12 month Sodium fluoride (G)		10 (corresponding to 4.5 mg F^–^/kg b.w./day)	Hemato (decreased leucocyte and hemoglobin levels)		4.5		Susheela and Jain (1983)

*Bd wt* body weight, *Cardio* cardiovascular, *DW* drinking water, *F* females, *G* gavage, *Gastro* gastrointestinal, *GW* gavage in water, *Hemato* hematological, *LOAEL* lowest-observed-adverse-effect level, *M* males, *Musc/skel* muscular/skeletal, *NaF* sodium fluoride, *NOAEL* no-observed-adverse-effect level, *Resp* respiratory

^a^As reported by the authors

**Table 2:** Selected high-quality developmental toxicity studies

Species, strain, number of animals	Exposure duration, chemical form, route	NaF concentration in DW (mg/L)	Doses (mg/kg/d)	NOAEL (mg/L) (mg/kg/d)	LOAEL (mg/L) (mg/kg/d)	Outcome (as stated by the authors)	Comments	References
Rat, CD, 26/group	GD 6 through 15 Investigations were done on GD 20 Sodium fluoride (DW)	< 0.6, 50, 150, 300	NaF^a^: 0, 6.6, 18.3, 27.1	≥ 300 mg/L NaF 27 mg NaF/kg/d^a^	–	Maternal exposure to NaF during organogenesis did not significantly affect the frequency of postimplantation loss, mean fetal body weight/litter, or external, visceral or skeletal malformations	NOAEL is the highest dose tested Drinking water: < 0.6 mg/L NaF Feed: 12.4 mg per kg fluoride	Heindel et al. (1996)
			Total F^–^ intake (water and feed)^a^: 1, 4.0, 9.3, 13.2	**13.2** mg F^–^ /kg/d^a^				
Rabbit, New Zealand White rabbits, 26/group	GD 6 through 19 Investigations were done on GD 30 Sodium fluoride (DW)	< 0.6, 100, 200, 400	NaF^a^: 0, 10.3, 18.1, 29.2	≥ 400 mg/L NaF 29 mg NaF/kg/d^a^	–	Maternal exposure to NaF during organogenesis did not significantly affect the frequency of postimplantation loss, mean fetal body weight/litter, or external, visceral or skeletal malformations	NOAEL is the highest dose tested Drinking water: < 0.6 mg/L NaF Feed: 15.6 mg per kg fluoride	Heindel et al. (1996)
			Total F^–^ intake (water and feed)^a^: 0.8, 5.8, 8.8, 13.7	**13.7** mg F^–^ /kg/d^a^				
Rat, CD, 35–37/group	GD 0 through GD 20 Investigations were done on GD 20 Sodium fluoride (DW)	0, 10, 25, 100, 175, 250	NaF^a^: 0, 1.4, 3.9, 15.6, 24.7, 25.1	175 mg/L NaF 24.7 mg NaF/kg/d^a^	250 mg/L 25.1 mg NaF/kg/d^a^	Fetal growth, number of external anomalies in fetuses, development of specific bones, including sternebrae were not affected by NaF A significant increase in the average number of fetuses with three or more skeletal variations was observed in the 250 mg/L group The number of litters with fetuses with three or more skeletal variations was increased in the 250 mg/L group (not statistically significant)	Water consumption in the 175- and 250-mg/L groups was significantly less than that of the control females. The daily amount of NaF ingested was less than expected at the two higher dose levels Feed: 7.95 mg/kg fluoride Control: Aqua Cool Ultra Pure water	Collins et al. (1995)
			F^–^^a^: 0, 0.6, 1.8, 7.1, 11.2, 11.4	**11.2** mg F^– ^/kg/d^a^	**11.4** mg F^–^ /kg/d^a^			
Rat, CD, 48/sex/group	Continuously during three generations. F0 rats were treated for 10 weeks and mated within groups Investigations of F0, F1 and F2 fetuses were done on GD 20 Sodium fluoride (DW)	0, 25, 100, 175 or 250	NaF^a^: 0, 3.4, 12.4–13.2, 18.8–19.3, 25.8–28.0	175 mg/L NaF 18.8–19.3 mg NaF/kg/d	250 mg/L NaF 25.8–28.0 mg NaF/kg/d	Sodium fluoride in drinking water at 175 mg/L produced no compound-related effects. Numbers of corpora lutea, implants, viable fetuses and fetal morphological development were similar in all groups. No dose-related anomalies in internal organs were observed in F2 fetuses Ossification of the hyoid bone of F2 fetuses was significantly decreased at 250 mg/L (considered as LOAEL)	Feed: 7.95 mg/kg fluoride Concentration of fluoride in the Pico system treated water: < 0.2 mg/L	Collins et al. (2001b)
			F^–^^a^: 0, 1.5, 5.6–6.0, 8.5–8-7, 11.7–12.7	**8.5–8.7** mg F^–^ /kg/d	**11.7–12.7** mg F^–^ /kg/d			

*DW* drinking water, *GD* gestation day, *NaF* sodium fluoride

^a^As reported by the authors of the study

**Table 3:** Animal studies published between 2005 and 2019 which investigated the effects of developmental fluoride exposure (without studies investigating neurobehavioral endpoints)

Species, strain, number of animals	Exposure duration, chemical form, route	NaF concentration in DW (mg/L)	Doses (mg/kg/d)	NOAEL (mg/L) (mg/kg/d)	LOAEL (mg/L) (mg/kg/d)	Outcome (as stated by the authors)	Limitations	References
Rat, Wistar *n *= 45 (F0) 7 dams/group	Multigenerational study: continuous exposure during three generations (F0, F1, F2) Sodium fluoride (DW)	1, 10, 50, 100	Dams: NaF^a^: 0.05, 0.5, 2.5, 5 F^–a^: 0.02, 0.23, 1.1, 2.3	10 mg/L NaF 0.5 mg NaF/kg/d 0.23 mg F^–^/kg/d	50 mg/L NaF 2.5 mg NaF/kg/d 1.13 mg F^– ^/kg/d	50 and 100 mg/L NaF (*n *= 7/group): significant histopathological changes were found in the myocardial tissue: myocardial cell necrosis, extensive cytoplasmic vacuole formation, nucleus dissolution in myosits, swollen and clumped myocardial fibers, fibrillolysis, interstitial edema, small hemorrhagic areas and hyperemic vessels	Blinding: NR Control for litter effects: NR Inadequate reporting of concentration in DW: unclear whether referring to NaF or F^–^ Group size < 10 F^–^ concentration in DW (control) and feed: NR	Cicek et al. (2005)
Mouse (species unclear), Wistar 10 dams/group	GD 15 until PND 14	500	NaF^b^: 75 mg/kg/d F^–b^: 34 mg/kg/d		500 mg/L NaF 75 mg NaF/kg/d 34 mg F^–^/kg/d	Fluoride given to dams led to oxidative stress in mothers as well as in offspring, able to induce enhanced lipid peroxidation levels and protein conformational changes, as suggested by stress protein (HSP, GRP) expression changes	Species/strain unclear Characterization of the test compound: NR Single high dose tested Blinding: NR Control for litter effects: NR Treatment duration < pre- and postnatal period F^–^ concentration in DW (control) and feed: NR	Bouaziz et al. (2007)
Rat, Wistar *n* = 6/group	Female rats throughout gestation and lactation, neonates received tap water until PND 90 Sodium fluoride (DW)	4.5 and 9.0	Dams: NaF^a^: 0.23, 0.45 F^–a^: 0.1, 0.2	–	4.5 mg/L NaF 0.23 mg NaF/kg/day 0.1 mg F^–^ /kg/day	Treatment groups: (*n *= 6). PND 90: male pups showed changes in sperm parameters (sperm count, viability) at 4.5 and 9 mg/L NaF	Blinding: NR 2 dose levels tested, no NOAEL established Group size < 10	Reddy et al. (2007)
Rat, Sprague-Dawley *n *= 40 (20/sex) *n* = 5/group	From pre-pregnancy to PND 56 Sodium fluoride (DW)	0, 25, 50, 100	Dams: NaF^a^: 0, 1.25, 2.5, 5 F^–a^: 0, 0.57, 1.13, 2.3	25 mg/L NaF 1.25 mg NaF/kg/day 0.57 mg F^–^/kg/day	50 mg/L NaF 2.5 mg NaF/kg/day 1.13 mg F^–^/kg/day	Testes of offspring at PND 56 (*n* = 5/group): effects of NaF on testicular histopathology, testicular ultrastructure and germ cell apoptosis at 50 and 100 mg/L NaF	Group size < 10 Control for litter effects: NR F^–^ concentration in DW (control) and feed: NR	Zhang et al. (2013)
Mouse, ICR, 6–8 weeks of age Number of animals unclear	3 treatment groups: Group I: male mice given 100 mg/L NaF in water for 35 days Group II: female mice mated with male mice not given NaF; 100 mg/L NaF in DW for 48 h after becoming pregnant Group III: control Sodium fluoride (DW)	100	NaF^a^: 9 F^–a^: 4.1	–	100 mg/L NaF 9 mg NaF/kg/day 4.1 mg F^–^ /kg/day	NaF treatment disrupted DNA methylation of H19 and Peg3 in early embryonic stages of the mouse. However, there were no significant changes in DNA methylation in sperm and liver of male mice treated with NaF	Single dose tested Blinding: NR Control for litter effects: NR Number of animals: NR Experimental setting not clearly described: e.g., discrepancy between description in methods and in the abstract/figures F^–^ concentration in DW (control) and feed: NR	Zhu et al. (2014)
Mouse, ICR, 6–8 weeks of age Number of animals during treatment unclear	4 treatment groups Group I: female mice mated with male mice not given NaF; 120 mg/L NaF in DW for 48 h after becoming pregnant Group II: male mice given 120 mg/L NaF in water for 35 days Group III: female mice mated with NaF-treated males, 120 mg/L NaF for 48 h after becoming pregnant Group IV: control Sodium fluoride (DW)	120	NaF^a^: 10.8 F^–a^: 4.9	–	120 mg/L NaF 10.8 mg NaF/kg/day 4.9 mg F^–^ /kg/day	120 mg/L NaF in drinking water of pregnant mice for 48 h: NaF affected DNA methylation of early embryos (*n* = 20). H19 gene was significantly downmethylated in embryos from pregnant mice. Methylation of both H19 and Peg3 genes was disrupted when the parent male mice were treated with NaF for 35 days	Single dose tested Blinding: NR Control for litter effects: NR Number of animals during treatment: NR F^–^ concentration in DW (control) and feed: NR	Zhao et al. (2015)
Rat, Sprague-Dawley	From pre-pregnancy to PND 56 Sodium fluoride (DW)	0, 25, 50, 100 Control: tap water (fluoride ion concentration < 1.0 mg/L)	Dams NaF^a^: 1.25, 2.5, 5 F^–a^: 0.57, 1.13, 2.3	–	25 mg/L NaF 1.25 mg NaF/kg/day 0.57 mg F^–^/kg/day	Testes of offspring were excised on PND 56 (*n *= 3/group): NaF exposure induced histological lesions in rat testes and caused extensive abnormalities in testicular tissue (all dose groups)	Group size unclear (start, pregnancy, treatment) Blinding: NR Control for litter effects: NR F^–^ concentration in feed: NR	Zhang et al. (2016)

Search criteria were: PubMed database for animal studies published between January 2005 and February 2019 with the key words in the title/abstract including ‘fluoride’, ‘sodium fluoride’ and ‘developmental toxicity’. In addition to the PubMed search, the reference lists of included studies and records that do not contain original data (i.e., reviews, editorials, or commentaries) were checked for further studies. Publications where the full text was not available in English or not published in a journal (master thesis, dissertations etc.) were excluded

*DW* drinking water, *GD* gestation day, *NaF* sodium fluoride, *NR* not reported, *PND* postnatal day

^a^Conversion of F^–^concentration in drinking water into daily doses was performed by application of default conversion factors derived by EFSA (EFSA 2012)

For chronic studies, a default factor of 0.05 for rats and 0.09 for mice should be used, e.g., 1 mg/L in water is equivalent to a dose of 0.05 and 0.09 mg/kg b.w. per day in rats and mice, respectively. For subchronic studies, a default factor of 0.09 for rats and 0.15 for mice should be used. For subacute studies, a default factor of 0.12 for rats and 0.18 for mice should be used

In case the authors only reported F^–^ concentration in drinking water, in the present estimation conversion factors of 0.05/0.09 were used for adult animals/the parental generation and conversion factors of 0.12/0.18 for the pups or short-term exposure of young animals

^b^as reported by the authors

**Table 4:** Neurobehavioral effects of fluoride in experimental animals since 2016 (not included in the NTP report, 2016)

Species, strain, number of animals	Exposure duration, chemical form, route	Concentration in DW (mg/L)	F^–^ Doses^a^ (mg/kg/d)	NOAEL F^–^ (mg/L) (mg/kg/d)	LOAEL F^–^ (mg/L) (mg/kg/d)	Outcome (as stated by the authors)	Limitations	References
**Developmental exposure**								
Rat, Sprague Dawley 40 F, 20 M (180–220g) 10 dams/dose	From pre-pregnancy until 2 months of delivery Sodium fluoride (DW)	NaF: 10, 50, 100 F^–^: 4.52, 22.6, 45.2 Control group: tap water, < 1.0 mg/L F^−^	Dams^a^: 0.23, 1.13, 2.26 Pups^a^: 0.54, 2.71, 5.42	4.52 mg/L Corresponding to^a^: Dams: 0.23 mg/kg/d Pups: 0.54 mg/kg/d	22.6 mg/L Corresponding to^a^: Dams: 1.13 mg/kg/d Pups: 2.71 mg/kg/d	50 mg/L NaF and higher caused learning and memory impairments (MWM task, *n *= 5)	Control for litter effects: NR F^–^ concentration in food: NR MWM: age of pups NR; influence of activity level/motor deficits NR	Zhao et al. (2019)
Rat, Long–Evans hooded timed-pregnant dams	Start at GD 6 continued to PND 90 Sodium fluoride (DW)	2 DW control groups: low F^–^ diet (3.24 mg/kg F^–^); standard diet (20.5 mg/kg F^–^) Treatment: 10, 20 mg/L F^–^ with low F^–^ diet	0.08, 0.16^b,c^	20 mg/L 0.16 mg/kg/d^b^	–	Tests were performed at different ages according to guidelines Male pups were used for testing (*n* = 10–22) No exposure-related differences in motor, sensory, or learning and memory performance No influence on thyroid hormone parameters No exposure-related pathology in the heart, liver, kidney, testes, seminal vesicles, or epididymis Mild inflammation in the prostate gland at 20 mg/L F^−^ When examined as adults (> PND90), rats in the 20 ppm F^−^ dose group showed evidence of mild fluorosis Significantly elevated internal F^–^ burden in the weanling brain and femur	Number of dams per dose: NR 2 dose levels tested, no LOAEL	McPherson et al. (2018)
Rat, Wistar 90–120 days old 10/group	Dams: exposure from GD 0 until PND 21 Sodium fluoride (DW)	Fluoride treated groups: 5, 10 mg/L in filtered tap water; Control group: filtered tap water	0.27 and 0.54^b^	–	5 mg/L (Long term memory retention) Corresponding to^b^: 0.27 mg/kg/d	Tests: female offspring at PND 90 Inhibitory avoidance test: 5 mg/L F: latency did not differ from that of control at the short-term memory retention test, but was significantly shorter at the long- term memory retention test (*p* < 0.05) 10 mg/L F: latencies of rats were significantly shorter in both short- and long-term memory retention (*p* < 0.05)	Concentrations in DW: not clear whether NaF or F^–^. Blinding: NR Conversion of DW concentration in doses: unclear F^–^ concentration in DW (control) and feed: NR Neurobehavioral tests: influence of activity level/motor deficits: NR 2 dose levels tested: no NOAEL	Bartos et al. (2018)
Rat, Sprague-Dawley 200 ± 20 g 10/group	From pregnancy until 6 months of delivery Sodium fluoride (DW)	NaF: 10, 50, 100 F^–^: 4.52, 22.6, 45.2 Control: < 1.0 mg/L fluorine	Dams^a^: 0.23, 1.13, 2.26 Pups^a^: 0.54, 2.71, 5.42	4.52 mg/L Corresponding to^a^: Dams: 0.23 mg/kg/d Pups: 0.54 mg/kg/d	22.6 mg/L Corresponding to^a^: Dams: 1.13 mg/kg/d Pups: 2.71 mg/kg/d	MWM test: significant effects at 50 and 100 mg/L NaF on memory and learning (e.g. escape latency and the swimming distance) Comment: effects partly showed no dose and time dependence	Control for litter effects: NR F^–^ concentration in feed: NR Neurobehavioral tests: influence of activity level/motor deficits NR, age of animals NR	Chen et al. (2018)
Mouse, ICR 8–10 weeks old *n *= 60, number of animals/group unclear	Parents: one month and during gestation and lactation Offspring: until PND 90 Chemical form not reported (DW)	NaF (not clear): 50, 100 F^–^: 22.6, 45.3 Control: 0 mg/L F^–^(analytics not reported)	Dams^a^: 2.0, 4.1 Pups^a^: 4.1, 8.2	–	22.6 mg/L Dams^a^: 2.0 mg/kg/d Pups^a^: 4.1 mg/kg/d	MWM test (*n *= 6/group): 50 and 100 mg/L fluoride significantly prolonged the escape latency period. The number of crossings in a particular zone was decreased but not significantly	Characterization of test compound: NR Concentration in drinking water: not clear whether NaF or F^–^ was used. Randomization: NR Blinding: NR Control for litter effects: NR F^–^ concentration in DW (control) and feed: NR Neurobehavioral tests: influence of activity level/motor deficits: NR	Ge et al. (2018)
Mouse, Kunming 48 adults (24 M; 24 F; 20–25 g each), *n* = 6/group	Start at GD 0 throughout lactation Sodium fluoride (DW)	NaF: 25, 50, 100 F^–^: 11.3, 22.6, 45.3		22.6 mg/L Corresponding to^a^: Dams: 2.0 mg/kg/d	45.3 mg/L Corresponding to^a^: Dams: 4.1 mg/kg/d	Neurobehavioral tests: offspring at PND 21 (*n* = 6) 100 ppm NaF: significantly enhanced number of total arm entries and working memory errors in the radial arm maze test compared to the control group. No difference was observed in open-field behaviors	Blinding: NR Control for litter effects: NR F^–^ concentration in DW (control) and feed: NR Neurobehavioral tests: influence of activity level/motor deficits: NR	Sun et al. (2018)
Mouse, ICR (25–30g) *n*/group unclear	from GD 7 to PND 21 Sodium fluoride (DW)?	NaF: 0, 25, 50, 100 F^−^: 0, 11.3, 22.6, 45.3 Control: analytics not reported	Dams: 0, 1.0, 2.0, 4.0	11.3 (learning and memory) Corresponding to^a^: Dams: 1.0 mg/kg/d	22.6 (learning and memory) Corresponding to^a^: Dams: 2.0 mg/kg/d	Neurobehavioral tests: offspring at PND 21 (*n* = 15) Open field test: number of entries into the center zone in 100 mg/L NaF group were significantly decreased No difference in the distance travelled and the time spent in center zone between control and F^–^ treatment groups was reported Eight-arm maze test: number of working memory errors, reference memory errors, and the total arm entries were significantly increased in 100 mg/L fluoride treatment group, but not on all training days. At 50 mg/L F^–^: significant effects on working memory error and number of total arm entries only on day 7	Characterization of test compound: NR Randomization: NR Blinding: NR Control for litter effects: NR F^–^ concentration in DW (control) and feed: NR Neurobehavioral tests: influence of activity level/motor deficits: NR Effects were not observed on all training days	Wang et al. (2018)
Rat, Wistar Albino Timed pregnant rats aged 160–180 days 6/group	53 days; gestational and post gestational period Sodium fluoride (DW)	NaF: 20 mg/L; 20 mg/L + quercetin: 20 mg/kg/d by gavage F^–^: 9.1 mg/L Control group: normal tap water	Dams^a^: 0.46		9.1 mg/L Corresponding to^a^: Dams: 0.46 mg/kg/d	Neurobehavioral tests: offspring at PND 14, 21, 30 Maze learning and open field: NaF treated pups showed significant (*p *< 0.05) decrease in learning ability, cognition, and increase in the latency period of goal achieving compared to the control Comment: preventive effect of quercetin was studied	NaF dose/concentration is unclear Blinding: NR Control for litter effects: NR Single dose tested F^–^ concentration in DW (control) and feed: NR Neurobehavioral tests: influence of activity level/motor deficits NR	Mesram et al. (2017)
**Adult exposure**								
Mouse, Kunming *n *= 60, 10/group	Three different exposure periods: 90, 120, 150 days Sodium fluoride (DW)	F^–^: 68 mg/L Control: deionized water	6.1	68 mg/L (90 day exposure) Corresponding to^a^: 6.1 mg/kg/d	68 mg/L (120 and 150 day exposure) Corresponding to^a^: 6.1 mg/kg/d	8 /group were tested for a)Anxiety NaF for 90 days: no significant changes compared to the control group NaF for 120 days: significant alterations in all tests (*p* < 0.05) NaF for 150 days: elevated zero maze and emergence test were significantly altered (*p* < 0.05) b)Depression-like behavior: Changes were significantly elevated in mice exposed to 120 days compared to control (*p* < 0.05) No significant alterations were observed among 90 and 150 days treatment groups Comment: results are inconsistent with regard to exposure duration	Blinding: NR Single dose tested F^–^ concentration in feed: NR Neurobehavioral tests: influence of activity level/motor deficits: NR	Li et al. (2019)
Rat, Wistar, M age of 5 weeks (80–110 g) 10/group	4 weeks or 12 weeks Sodium fluoride (DW)	F^–b^: 0, 60, 120	3.0, 6.0^a^	60 mg/L (MWM) Corresponding to^a^: 3.0 mg/kg/d (MWM)	120 mg/L (MWM) Corresponding to^a^: 6.0 mg/kg/d 60 mg/L (open field test) Corresponding to^a^: 3.0 mg/kg/d (MWM)	12 weeks of exposure, MWM at 120 mg/L: latency time was significantly longer than that of control group on the 1st and 2nd days of training sessions (*p* < 0.05), but not on 3rd to 5th day Open field test: number of instances of standing significantly decreased in all treated rats as compared to the control (*p* < 0.05). Numbers of crossing between center and surround zone significantly decreased in the high dose group. No differences for the distance moving in the center and the time spent in the center area among the different experimental groups Dental fluorosis was obvious in all treated rats Comment: results unclear: dependent on the training day	Concentration in DW is unclear: NaF or F^–^. F^–^ concentration in DW (control) and feed: NR Neurobehavioral tests: influence of activity level/motor deficits NR	Yang et al. (2018)
Rat, Wistar, M 8/group	30 days Sodium fluoride Administration orally via intra-gastric tube		NaF^b^: 5, 10, 20 F^–^: 2.3, 4.5, 9.1 Control: water with low F^–^ level (< 0.027 mg/L)	9.1 mg /kg/d		Y-maze (working memory test): No significant difference in working memory was reported among groups There were significant increases in the degree of fluorosis during the treatment	Exposure route: not via DW F^–^ concentration in feed: NR 3 dose levels tested, no LOAEL	Pulungan et al. (2018)
Rat, Sprague-Dawley One-month old (100–120 g) 10/group	10 months Sodium fluoride (DW)	50 mg/L (DW); 50 mg/L fluoride (DW) + 50 mg vitamin E/kg/d by intragastric administration Untreated Control (< 0.5 mg/L F^–^)	2.5		50 mg/L Corresponding to^a^: 2.5 mg/kg/d	MWM: at 50 mg/L increased escape latency time, decreased number of crossings of the platform site, decreased time of staying on the site of the platform F^–^ concentration in feed: normal diet containing < 6 mg/kg fluoride Comment: preventive effects of vitamin E were examined	Characterization of the test compound: unclear whether NaF or F^-^concentration in DW Single dose tested Neurobehavioral tests: influence of activity level/motor deficits NR	Dong et al. (2017)
Mouse, Swiss Albino, M One month old (30 ±5 g) 7/group	30 days Sodium fluoride (DW)	NaF: 120 mg/L; 120 mg/L + Curcumin or Resveratrol (30 mg/kg/d, orally) F^–^: 54.4 mg/L Control: F^–^free RO water (analytics not reported)	4.9		54.4 mg/ Corresponding to^a^: 4.9 mg/kg/d	120 mg/L NaF: influence on learning and memory (MWM, CM test) Comment: preventive effect of curcumin and resveratrol was studied	Randomization: NR Blinding: NR Single dose tested F^–^ concentration in DW (control) and feed: NR Neurobehavioral tests: detailed description of the test was missing, influence of activity level/motor deficits NR	Sharma et al. (2018b)

Search criteria were: PubMed database for animal studies published between January 2005 and February 2019 with the key words in the title/abstract including ‘fluoride’, ‘sodium fluoride’ and ‘neurotoxicity’ and/or ‘developmental toxicity’ and/or ‘brain’. In addition to the PubMed search, the reference lists of included studies, records that do not contain original data (i.e., reviews, editorials, or commentaries), and the Fluoride Action Network website (https://fluoridealert.org/studies/brain02/) were checked for further studies. Publications where the full text was not available in English or not published in a journal (master thesis, dissertations etc.) were excluded

*CM* classic maze, *DW* drinking water, *F* female, *GD* gestation day, *M* male, *MWM* morris water maze, *NR* not reported, *PND* postnatal day

^a^Conversion of F^–^concentration in drinking water into daily doses was performed by application of default conversion factors derived by EFSA (EFSA 2012). For chronic studies, a default factor of 0.05 for rats and 0.09 for mice should be used, e.g., 1 mg/L in water is equivalent to a dose of 0.05 and 0.09 mg/kg bw per day in rats and mice, respectively. For subchronic studies, a default factor of 0.09 for rats and 0.15 for mice should be used. For subacute studies, a default factor of 0.12 for rats and 0.18 for mice should be used. In case the authors only reported F^–^ concentration in drinking water, conversion factors of 0.05/0.09 (rats/mice) were used for adult animals/the parental generation and conversion factors of 0.12/0.18 (rats/mice) for the pups or short-term exposure of young animals

^b^As indicated by the authors

^c^Values obtained by applying default conversion factors of EFSA (EFSA 2012): dams (conversion factor of 0.05): 0.5, 1 mg/kg/d; pups (conversion factor of 0.12): 1.2, 2.4 mg/kg/d

**Table 5:** Reproductive toxicity in experimental animals (selected high-quality studies)

Species, strain, number of animals	Exposure duration, chemical form, route	NaF-concentration in DW (mg/L)	Doses (mg/kg/d)	NOAEL (mg/L) (mg/kg/d)	LOAEL (mg/L) (mg/kg/d)	Outcome (as stated by the authors)	Comment	References
Rat, CD, 48/sex/group	Continuously during three generations F0 rats were treated for 10 weeks and mated within groups Investigations of F0, F1 and F2 fetuses at GD 20 Sodium fluoride (DW)	0, 25, 100, 175, 250	NaF^a^: 0, 2.8–3.8, 11.0–14.6, 18.0–21.8, 23.1–28.0 0, F^–a^: 1.3–1.7, 5.0–6.6, 8.1–9.9, 10.5–12.7	250 mg/L NaF 23.1–28.0 mg NaF/kg/d 10.5–12.7 mg F^–^/kg/d	–	NaF up to 250 mg/L did not affect reproduction in rats. No cumulative effects were observed in the three generations Mating, fertility and survival indices were not affected	Diet: NIH-07 low fluoride (7.95 mg/kg F^–^) Concentration of F^–^ in the Pico system treated water: < 0.2 mg/L	Collins et al. (2001a)
Rat, CD, 48/sex/group	Continuously during three generations F0 rats were treated for 10 weeks and mated within groups Investigations of F0, F1 and F2 fetuses at GD 20 Sodium fluoride (DW)	0, 25, 100, 175, 250	NaF^a^: 0, 2.8–3.0, 11.0–11.3, 18.0–18.6, 23.1–24.1 F^–a^: 0, 1.3–1.4, 5.0–5.1, 8.1–8.4, 10.5–10.9	250 mg/L NaF 23.1–24.1 mg NaF/kg/d 10.5–10.9 mg F^–^/kg/d	–	Effects on male reproduction: prolonged exposure to NaF in drinking water up to 250 mg/L did not adversely affect spermatogenesis or endocrine function in the F0 or F1 generation of male rats	Diet: NIH-07 low fluoride (7.95 mg/kg F^–^) Concentration of F^–^ in the Pico system treated water: < 0.2 mg/L	Sprando et al. (1997, 1998)

*DW* drinking water, *GD* gestation day, *NaF* sodium fluoride

^a^As reported by the authors of the study

### Chronic toxicity

A number of chronic toxicity studies that focused on systemic effects resulted in LOAELs in rats, mice, and rabbits ranging between 4.3 and 7.6 mg/kg b.w./day (Table 1). Adverse effects were observed in the respiratory, cardiovascular, gastrointestinal, hematological, hepatic, renal, and muscular/skeletal system, showing that fluoride can cause a wide range of systemic effects at the tested doses (Table 1). Interestingly, the NOAELs and LOAELs derived from the chronic toxicity studies were relatively similar among the three species.

### Developmental toxicity

A comprehensive summary of studies published between 1990 and 2005 is provided in the National Research Council (NRC) report, demonstrating that developmental processes are susceptible to fluoride (NRC 2006). Four developmental toxicity studies are highlighted, because of their compliance to standard guidelines, adequate numbers of animals, and administration of sodium fluoride in drinking water. These studies resulted in NOAELs of 13.2 mg/kg b.w./day for rats (Heindel et al. 1996), 13.7 mg/kg b.w./day for rabbits (Heindel et al. 1996), 11.2 mg/kg b.w./day for rats (Collins et al. 1995), and 8.5–8.7 mg/kg b.w./day for rats (Collins et al. 2001b); (Table 2). While Heindel et al. (1996) found no adverse effects at doses up to 13.7 mg/kg b.w./day, Collins et al. reported a significant increase in the average number of fetuses with three or more skeletal variations at a dose of 11.4 mg/kg b.w /day (Collins et al. 1995) or decreased ossification of the hyoid bone of F2 fetuses at 11.7 mg/kg b.w./day (Collins et al. 2001b).

Since the report of the NRC, no further developmental studies conducted according to standard guidelines (OECD or NTP) have become available. A literature search from 2005 to 2018 revealed a number of animal studies that reported an effect of fluoride exposure during development on various end points in offsprings (excluding neurobehavioral effects which will be discussed in a separate paragraph), e.g., histopathological changes in the myocardial tissue (Bouaziz et al. 2007), induction of oxidative stress (Cicek et al. 2005), histological lesions in testes, and abnormalities in testicular tissue (Zhang et al. 2013, 2016), and an influence on sperm parameters (Reddy et al. 2007). In part, these end points, e.g., the induction of oxidative stress or histopathology of the myocardium are not covered by the standard guidelines. Furthermore, fluoride exposure was shown to alter DNA methylation in early mouse embryos (Zhao et al. 2015; Zhu et al. 2014). The respective studies are quite heterogeneous with respect to experimental settings (e.g., animal model, dose levels) and end points examined. Overall, all studies reported lower NOAELs (0.23–0.57 mg/kg/day) and LOAELs (0.1–4.9 mg/kg/day) than the above summarized studies (Table 3). If the authors only reported fluoride concentrations in drinking water, the conversion into daily doses was performed by applying default conversion factors that were derived by EFSA (EFSA 2012) (Table 3). A review of the quality of these studies identified various limitations that hamper their interpretation and reduce their value for risk assessment. For example, the following aspects were often not adequately addressed:

(i) Characterization of the test compound, e.g., source, purity, and chemical form of fluoride
(ii) Randomization
(iii) Blinding of treatment and outcome assessment
(iv) Key study information, e.g., species/strain, gender, or number of animals used for treatment and composition of the animal die
(v) Experimental setting, e.g., outcome assessment, number of dose levels, and duration of treatment
(vi) Control for litter effects

Furthermore, only a single high dose was investigated in some studies, which was identified as the LOAEL; therefore, no dose-response assessment was possible (Bouaziz et al. 2007; Zhao et al. 2015).

The group size in the studies was low, ranging from 5 to 10 animals whereas according to the respective standard guidelines, group sizes of more than 20 are required (e.g., OECD Guideline 426). The limitations of each single study are listed in Table 3. Overall, some of the studies could be used to gain initial mechanistic information, but were not appropriate to perform a dose-response assessment for developmental toxicity, or to derive a point of departure (POD) and an MoE. In the future, it will be important to clarify whether the observed lower LOAELs are valid by conducting standardized, quality-controlled studies. Furthermore, studies are also required that provide quantitative measures, such as effect size, POD, identification of a NOAEL, and a LOAEL dose, and parameters for a benchmark analysis [summarized by (NTP 2016)].

### Neurobehavioral studies and neurotoxicity

Prior to the 2006 NRC review, few animal studies reported alterations in the behavior of rodents after treatment with fluoride (NRC 2006). According to NRC, the observed changes were judged to be not substantial in magnitude and it was stated that they could have been due to alterations in hormonal or peptide activity (NRC 2006). In recent years, numerous studies on the potential neurotoxicity of fluoride in experimental animals have been published. Detrimental effects on behavior in animal studies, including prioritizing assessment of learning and memory outcomes, have recently been reviewed by the National Toxicology Program (NTP 2016). Thirty percent of the studies identified were excluded from the systematic review due to concerns of bias, primarily because of at least three of the limitations already mentioned above. Of note, very few of the remaining studies assessed effects on learning and memory resulting from exposure to fluoride levels of approximately 0.7 mg/L—the recommended level for community water fluoridation in the USA (NTP 2016). Several studies suggested performance deficits in learning and memory tasks in rats when fluoride levels exceeded 100 mg/L in the drinking water (NTP 2016). A number of studies also reported such effects in rats at 2–50 mg/L fluoride [corresponding to 0.1–2.5 mg/kg b.w./day applying EFSA default conversion factors (EFSA 2012)]. However, many of these findings occurred in the presence of motor dysfunction or general toxicity, thus diminishing confidence in any conclusion regarding learning deficits (McPherson et al. 2018; NTP 2016). Overall, the systematic review by the NTP reported a low to moderate level of evidence for adverse effects on learning and memory in exposed animals with fluoride concentrations substantially higher than 0.7 mg/L (NTP 2016). Evidence is strongest (moderate level of evidence) in exposed adult animals, and weakest (low level of evidence) in animals exposed during development (NTP 2016).

After the publication of the NTP report in 2016, several studies have been published, which investigated the impact of fluoride exposure on memory and learning in experimental animals (Table 4). Here, we differentiated between studies in animals exposed during pre- and postnatal development to those exposed as adolescents/young adults and reviewed the quality of these studies. Risk of bias among individual neurobehavioral studies was estimated following similar criteria as applied by the NTP (NTP 2016), taking also into account the relevant OECD guidelines (e.g., OECD test no. 426, developmental neurotoxicity study); (OECD 2007). We assumed that missing information (concerning, e.g., randomization, blinding, controlling for litter effects) was an indication that the respective aspect was not considered.

The following four key requirements were considered essential to achieve adequate and comparable study quality (as summarized by NTP 2016): randomization of treatment group, blinding during neurobehavioral outcome assessment, adequate characterization of the administered chemical, and controlling for litter effects (NTP 2016). According to NTP, randomization and blinding during outcome assessment are considered as particularly critical factors for risk of bias assessment (NTP 2016). There is empirical evidence that failure to apply these factors can bias results away from the null hypothesis toward larger effects (NTP 2016; OECD 2007). Lack of blinding at outcome assessment is attenuated if behavioral measurements are performed by an automated, computer-driven system (NTP 2016). In addition, concern for lack of blinding during allocation or the conduction of the study can be reduced if blinding was carried out at outcome assessment (NTP 2016). Adequate characterization of the test compound is essential to assess the purity and stability of the chemical exposure (NTP 2016). Independent confirmation of purity would be considered best practice, since impurities may be more toxic than the chemical of interest (NTP 2016). However, an analytical verification was not performed for any of the studies presented in Table 4 before the start of exposure. Finally, when littermates are evaluated in developmental studies, control for litter effects is essential (NTP 2016; OECD 2007). Studies should consider that pups from a single litter may respond more similarly to one another compared to pups from different litters (NTP 2016).

Eight studies published since 2017 investigated the effect of fluoride on learning and memory following exposure during development (Bartos et al. 2018; Chen et al. 2018a; Ge et al. 2018; McPherson et al. 2018; Mesram et al. 2017; Sun et al. 2018; Wang et al. 2018; Zhao et al. 2019); and five additional studies examined exposure during young adulthood (Dong et al. 2017; Li et al. 2019; Pulungan et al. 2018; Sharma et al. 2018a; Yang et al. 2018). Only one study among them was conducted according to the generally accepted guidelines considering all of the aforementioned key requirements (McPherson et al. 2018). This recent study from the NTP laboratories was designed to address limitations identified in the NTP systematic review (McPherson et al. 2018; NTP 2016), and used exposure levels near the recommended level for community water fluoridation in the USA. For this purpose, equivalent human daily water intake of 1.74 mg fluoride/day for an adult, or 0.63–1.23 mg/day for children 1 to 14 years of age (EPA 2010) were approximated in rodents by using drinking water concentrations of 7–9 mg/L fluoride (NTP 2016). The highest dose of 20 mg/L fluoride was selected based upon the US EPA’s maximum contaminant level of 4 mg/L, based on the assumption that approximately fivefold higher doses are required for rats to achieve serum concentrations similar to those in humans (Dunipace et al. 1995; McPherson et al. 2018; NRC 2006). Pregnant Long-Evans hooded rats received a standard diet (20.5 mg/kg fluoride) or a low fluoride diet (3.24 mg/kg fluoride) with drinking water containing 0, 10, or 20 mg/L fluoride from gestational day 6 throughout lactation. Male pups were exposed throughout adulthood and underwent neurobehavioral testing using different paradigms to assess learning and memory (NTP 2015). No exposure-related differences in motor, sensory, or learning and memory performance (running wheel, open-field activity, light/dark place preference, elevated plus maze, pre-pulse startle inhibition, passive avoidance, hot-plate latency, Morris water maze (MWM) acquisition, probe test, reversal learning, and Y-maze) were observed with either of the two exposure levels investigated (McPherson et al. 2018). Therefore, a LOAEL and a dose-response assessment could not be established. In addition, there was no influence on thyroid hormone parameters (serum triiodothyronine (T3), thyroxine (T4), and thyroid stimulating hormone (TSH)). With the exception of mild inflammation in the prostate gland at 20 mg/L fluoride, no exposure-related pathology was observed in the heart, liver, kidney, testes, seminal vesicles, or epididymis (McPherson et al. 2018). Histological examination of the brain also revealed no evidence of neuronal death or glial activation in the hippocampus at the highest dose tested (McPherson et al. 2018). One further study which adequately considered the key requirements (Table 4) did not reveal any significant difference in working memory up to a fluoride dose of 9 mg/kg b.w./day (Pulungan et al. 2018). However, exposure was via an intragastric tube, and thus not representative of normal drinking water consumption. Three doses were investigated, none of which had an effect on memory and learning; therefore, a LOAEL and a dose-response relationship could not be established.

Some reports suggest that NaF has an effect on learning and memory. Six studies in rats (Bartos et al. 2018; Chen et al. 2018a; Dong et al. 2017; Mesram et al. 2017; Yang et al. 2018; Zhao et al. 2019) and five in mice (Ge et al. 2018; Li et al. 2019; Sharma et al. 2018a; Sun et al. 2018; Wang et al. 2018) reported such effects (mainly by investigating maze performance, e.g., MWM) with 20–120 mg/L NaF (9–54 mg/L fluoride) in drinking water corresponding to doses of approximately 0.46–6.1 mg/kg b.w./day. However, these studies had several limitations: two did not give adequate information with regard to the four key requirements (Ge et al. 2018; Wang et al. 2018). The overall reporting of data was insufficient and indicative of a high risk of bias. The remaining studies also revealed one to three of the key limitations (Table 4), e.g., lack of reporting of blinding (Bartos et al. 2018; Li et al. 2019; Mesram et al. 2017; Sharma et al. 2018a; Sun et al. 2018) or randomization (Sharma et al. 2018a), lack of controlling for litter effects (Chen et al. 2018a; Mesram et al. 2017; Sun et al. 2018; Zhao et al. 2019), or the lack of a clear characterization of the test compound, i.e., whether concentrations or dose levels refer to fluoride or sodium fluoride (Bartos et al. 2018; Dong et al. 2017; Mesram et al. 2017; Yang et al. 2018). Furthermore, only a single dose was investigated in several studies (Dong et al. 2017; Li et al. 2019; Mesram et al. 2017; Sharma et al. 2018a), which was identified as the LOAEL. In most of the studies, only single parameters were explored, which also is considered to be a study limitation. The group size in almost all studies was low, ranging from five to ten animals and did not meet the requirements of the relevant guidelines. Nevertheless, some of the findings, e.g., by Zhao et al. (2019), were quite consistent concerning neurobehavioral and neuropathological outcomes, thus requiring further investigations. They reported that 22.6 mg/L fluoride in drinking water (corresponding to approximately 2.7 mg/kg b.w./day fluoride, Table 4) caused learning and memory impairments (MWM test) in pups, which were accompanied by mitochondrial morphological alterations in the hippocampus manifested as fission suppression and fusion acceleration, along with defective autophagy, excessive apoptosis, and neuronal loss (Zhao et al. 2019).

Fluoride exposure originates from multiple sources (NTP 2016). However, information on alternative sources, such as food or water supply, was lacking in the majority of the aforementioned studies. When reported, fluoride levels in control water ranged from 0.03 to less than 1.0 mg/L and in rodent feed from 3.2–20.5 mg/kg. Furthermore, the majority of the studies investigated high doses of fluoride, which are not relevant for human exposure. Most studies examining the effects of fluoride exposure on learning and memory have only investigated avoidance conditioning or maze performance, both of which can be influenced by general activity level or motor deficits, thus limiting the ability to accurately evaluate a specific effect of fluoride on these parameters (NTP 2016).

The NTP review focused on selected behavioral measures, and did not include studies examining the effect of fluoride exposure on brain-related cellular, morphometric, or histological end points, or its influence on thyroid function, which may alter specific neurobehavioral measures (NTP 2016). These end points, last reviewed by the NRC (NRC 2006), provided evidence that fluoride interferes with brain and other physiological functions by both direct and indirect means (NRC 2006). However, the observed changes may be subtle or seen only under certain physiological or environmental conditions (NRC 2006). Potential mechanisms described by the NRC included reduced brain content of lipids and phospholipids, and enzymes that metabolize them, such as phosphohydrolases and phospholipase D, as well as the inhibition of cholinesterase activity (including acetylcholinesterase) and a reduction of acetylcholine (NRC 2006).

Since publication of the NRC report in 2006, numerous brain-related histological, chemical, and molecular studies have been conducted, and approximately 130 studies were identified from 2007 to 2019. A complete analysis of these studies was not within the scope of the present review, but importantly, some of the more recent studies that focused on neurobehavioral effects also addressed the histology/biochemistry underlying putative effects of fluoride. The results of these more recent studies are quite diverse, which may be due to different experimental setups (e.g., test concentrations, species/strain and age of the animals, exposure duration), but also due to several limitations (see Table 4). Most of the newer studies appear to support the conclusions of the NRC report (NRC 2006); however, some contradict findings of previous studies showing that fluoride at lower concentrations could interfere with brain functions. For example, a histological examination of the brain revealed no evidence of neuronal death or glial activation in the hippocampus upon exposure to 20 mg/L fluoride in drinking water (McPherson et al. 2018). There was also no significant difference in the number of pyramidal neurons in the medial prefrontal cortex cells after administration of 5, 10, and 20 mg/kg b.w./day of oral NaF solution (Pulungan et al. 2018). Other studies observed neuronal death or dysfunction in the rat hippocampus at concentrations of 10–120 mg/L NaF in drinking water (Basha et al. 2011; Chen et al. 2018a; Sharma et al. 2018a; Shashi and Kumar 2016; Teng et al. 2018; Wang et al. 2018; Yan et al. 2016; Zhao et al. 2019), an influence on neurotransmitter signaling (Dong et al. 2017; Sun et al. 2018), and induction of oxidative stress in the brain (Bartos et al. 2018; Dong et al. 2017). Further studies also reported stimulation of microglia following fluoride exposure (Shuhua et al. 2012; Yan et al. 2013, 2016; Yang et al. 2018). Factors accounting for the differences in the neuropathological findings between studies include differences in fluoride levels in food and the water source, as well as potential processing artifacts for neuronal death, e.g., the presence of contracted, intensely stained neurons (so-called ‘dark neurons’) which can be produced by postmortem manipulation or trauma in brain tissue (Jortner 2006; McPherson et al. 2018).

### Reproductive toxicity

A large number of studies evaluated the reproductive tract structure or function in animal models, primarily for the purpose of hazard identification using high doses of fluoride to reveal potentially sensitive reproductive tract targets and pathways (NRC 2006). The NRC, in its 2006 report, summarized some representative examples to provide an overview of the conclusions drawn from these studies (NRC 2006): (1) cessation of spermatogenesis and alterations in the epididymis and vas deferens were observed in rabbits administered NaF at 10 mg/kg b.w./day for 29 months (Susheela and Kumar 1991); (2) effects on Leydig cells and decreased serum testosterone were observed in rats exposed to NaF at 10 mg/kg b.w./day for 50 days (Narayana and Chinoy 1994); and (3) decreased activity of the steroidogenic enzymes (3β-hydroxysteroid dehydrogenase [HSD] and 17β-HSD) was found in the ovary and uterus of mice treated with NaF at 10 mg/kg b.w./day for 30 days (Chinoy and Patel 2001). In general, these studies show adverse effects of fluoride on the reproductive tract at concentrations sufficiently high to also induce other signs of toxicity.

A comprehensive multigenerational study of the effects of fluoride on reproduction has been conducted in rats using standard guidelines and the adequate numbers of animals (Collins et al. 2001a) (Table 5). Rats were administered drinking water with NaF at 0, 25, 100, 175, and 250 mg/L for three generations. No compound-related effects were found on mating or fertility, gestation or lactation, F1 survival, development, or organ weight. There were also no alterations to teeth, except for mild whitening observed in rats exposed to fluoride at 100 mg/L or greater. This well-conducted study concluded that NaF at concentrations of up to 250 mg/L in drinking water, which corresponds to a fluoride dose of 10.5–12.7 mg/kg/day, did not alter reproduction in rats (Collins et al. 2001a; NRC 2006). Since 2006, there have been numerous studies published investigating the effects of fluoride exposure during adolescence/adulthood on various reproductive parameters; however, a detailed analysis of these studies was beyond the scope of the present review.

## Epidemiological studies: does fluoride act as a human developmental neurotoxicant?

In recent years, reviews have been published that cite epidemiological studies that are supportive of the view that ‘normal fluoride exposure’ (via drinking water, dietary intake, toothpaste etc.) is harmful to humans; fluoride has also been categorized as a developmental neurotoxicant (Duan et al. 2018; Grandjean and Landrigan 2014; Nakamoto and Rawls 2018). A widely recognized and discussed example is the review by Grandjean and Landrigan published in *Lancet Neurology* (Grandjean and Landrigan 2014).

In this publication, the authors cited one of their previous studies, a meta-analysis from 2012 of 27 cross-sectional studies investigating children exposed to fluoride in drinking water (Choi et al. 2012). There, a decreased IQ was observed in ‘fluoride exposed’ compared to ‘reference populations’. However, Choi et al. (Choi et al. 2012) also discussed limitations of their findings, e.g., that critical confounders were not considered and age adjustment of cognitive test scores were not reported in most studies included in the meta-analysis. Nevertheless, in the *Lancet Neurology* review (Grandjean and Landrigan 2014), the authors concluded that fluoride is a human developmental neurotoxicant, although no novel data and arguments were presented. Moreover, it was stated that ‘confounding from other substances seems unlikely in most of these studies’ (Grandjean and Landrigan 2014) without supporting this statement with data. Besides this questionable reinterpretation, further limitations of the meta-analysis have already been discussed in detail by other authors (Feldman 2014; Gelinas and Allukian 2014; Sabour and Ghorbani 2013; Sutton et al. 2015), e.g., the use of non-validated IQ tests (Feldman 2014), exposure of the children to a relatively highly polluted environment, the subsequent risk of possible confounding substances (Feldman 2014; Gelinas and Allukian 2014), and an overall low quality of the meta-analysis (Sutton et al. 2015). Moreover, in the time period after the introduction of fluoridation of drinking water, IQs in general have increased (Feldman 2014). This may be due to secondary factors, such as improved education.

### Epidemiological studies since 2012

To reach a better understanding of potential associations between fluoride exposure and human intelligence, we conducted a literature search of epidemiological studies published between January 2012 and August 2019. A compilation of the 23 epidemiological studies identified is given in Table 6. Twenty studies were conducted with a cross-sectional design, one with a longitudinal (Bashash et al. 2017), and two with a prospective design (Broadbent et al. 2015; Green et al. 2019). The main analyzed endpoint was IQ in 22 of 23 studies, with one study examining an association between fluoride exposure and school performance (Mustafa et al. 2018). Study locations were: 13 in India, 4 in China, 2 in Iran, 1 in Sudan, 1 in Mexico, 1 in Canada, and 1 in New Zealand. All studies investigated human intelligence in children and adolescents, between the ages of 3 and 14 years. One study additionally considered intelligence in adults (Broadbent et al. 2015). Twenty-one of the 23 studies concluded that higher fluoride exposure was associated with lower intelligence. In contrast, two studies did not observe any association (Broadbent et al. 2015; Sharma et al. 2018c).

**Table 6:** Studies on a possible association of fluoride exposure from drinking water and human intelligence published since 2012

Reference	Study location	Study type (CS/PC/L)	Fluoride resource (E/CWF) content (mg/L); location of measurement	Comparator	Participant number [age (years)]	Statistical adjustment performed	Outcome	Results/conclusion, as stated by the authors
Saxena et al. (2012)	India (Madhya Pradesh State)	CS	E < 1.5 vs. 1.5–3.0 vs. 3.1–4.5 vs. > 4.5; water samples from each child’s home	IQ and water fluoride IQ and urinary fluoride Urinary fluoride and water fluoride	120 (12)	Yes	↓	Reduction in intelligence was observed with an increased water fluoride level (*p* 0.000). The urinary fluoride level was a significant predictor for intelligence (*p* 0.000). Children in endemic areas of fluorosis are at risk for impaired development of intelligence
Seraj et al. (2012)	Iran (Makoo)	CS	E 0.8 (± 0.3) vs. 3.1(± 0.9) vs. 5.2 (± 1.1); groundwater samples distributed over the study area	IQ and water fluoride	293 (6–11)	Yes	↓	Children residing in areas with higher than normal water fluoride levels demonstrated more impaired development of intelligence. Thus, children’s intelligence may be affected by high water fluoride levels
Nagarajappa et al. (2013)	India (Kutch District, Gujarat)	CS	E 0.5 vs. 2.4–3.5; data obtained from Water and Sanitation Management Organization, Gujarat	IQ and water fluoride	100 (8–10)	No	↓	Chronic exposure to high levels of fluoride in water was observed to be associated with lower intelligence quotient
Pratap et al. (2013)	India (Dausa district, Rajasthan)	CS^a^	E 1.03 (± 0.15) vs. 6.8 (± 1.6); water samples from each child’s home	IQ and water fluoride Urinary/serum fluoride and water fluoride	142 (9–12)	No	↓	IQ scores and serum fluoride levels was directly correlated with the concentration of serum fluoride level. The conclusion of the study is that the excessive fluoride delineates the neuronal impairment which were evident by reduced IQ score and serum acetylcholinesterase activity
Wei et al. (2014)	China (Bijie City, Guizhou Province)	CS^a^	E endemic fluorosis area (long-term treatment group vs. short-term treatment) vs. non-fluorosis area^b,c^	IQ and urinary fluoride	741 (8–12)	No	↓	Urinary fluoride was negatively correlated with the level of IQ (*r*=− 0.553, *p *< 0.01). The intelligence development of children in coal-burning-borne endemic fluorosis area is significantly delayed
Karimzade et al. (2014)	Iran (West Azerbaijan)	CS^a^	E 0.25 vs. 3.94; water samples from drinking water supplies (wells and springs) in the two study regions	IQ and water fluoride	39 (9–12)	No	↓	The study found that children residing in a region with a high drinking water fluoride level had lower IQs compared to children living in a low drinking water fluoride region (*p *< 0.001)
Broadbent et al. (2015)	New Zealand (Dunedin)	PC^d^	CWF vs. E 0.7–1.0 vs. 0.0–0.3 and/or fluoride dentifrice and/or intake of 0.5 mg fluoride tablets assessed in early life; with or without CWF coded from residential address data	IQ and water/dentifrice/tablets fluoride	992/942 (7–13/38)^e^	Yes	→	No clear differences in IQ because of fluoride exposure were noted. These findings held after adjusting for potential confounding variables
Choi et al. (2015)	China (Southern Sichuan)	CS^a^	E 1.0–4.07 (GM 2.20); data measured and recorded by Mianning County Center for Disease Control obtained from well-water in the communities	IQ and water fluoride IQ and urinary fluoride Urinary fluoride and water fluoride	51 (6–8)	Yes	↓	This pilot study in a community with stable life-time fluoride exposures supports the notion that fluoride in drinking water may produce developmental neurotoxicity, and that the dose-dependence underlying this relationship needs to be characterized in detail
Zhang et al. (2015a)	China (Tianjin City)	CS	E 1.40 (1.23–1.57) vs. 0.63 (0.58–0.68); water samples from each child’s home	IQ and water fluoride Urinary/serum fluoride and water fluoride IQ and urinary/serum fluoride	180 (10–12)	Yes	↓	Significantly high levels of fluoride in drinking water, serum, urine, along with poor IQ scores were observed in the high fluoride exposure group compared with those in control (all *p *< 0.05). Levels of fluoride serum and urine were inversely related with IQ (*r*^s^ = − 0.47, *p *< 0.01; *r*^s^ = − 0.45, *p *= 0.002)
Khan et al. (2015)	India (Lucknow district)	CS	E 0.19 vs. 2.41; water samples were collected from borewells	IQ and water fluoride	429 (6–12)	No	↓	Findings of this study suggest that the overall IQ of the children exposed to high fluoride levels in drinking water and hence suffering from dental fluorosis were significantly lower than those of the low fluoride area
Sebastian and Sunitha (2015)	India (Mysore District)	CS	E 0.4 vs. 1.2 vs. 2.2; information from Rajiv Gandhi National Rural Drinking Water Program	IQ and water fluoride	405 (10–12)	Yes	↓	In bivariate analysis, significant relationships were found between water fluoride levels and IQ of school children (*p *< 0.05) School children residing in area with higher than normal water fluoride level demonstrated more impaired development of intelligence when compared to school children residing in areas with normal and low water fluoride levels
Kundu et al. (2015)	India (Delhi)	CS	E high vs. low fluoride area^f^; water samples were collected from borewell	IQ and water fluoride	200 (8–12)	Yes	↓	Fluoride in the drinking water was significantly related with the IQ of children. Along with fluoride, mother’s diet during pregnancy was also found to be significantly related with IQ of children.
Aravind et al. (2016)	India (Virajpet, Banavara, Mastihalli)	CS	E < 1.2 vs. 1.2–2 vs. > 2^g^; drinking water samples from, e.g., open well/bore well/tube well	IQ and water fluoride	288 (10–12)	No	↓	It is concluded that IQ level was negatively correlated with fluoride level in drinking water
Das and Mondal (2016)	India (West Bengal)	CS	E 0.25–9.40 (M 2.11); water samples from tube wells of study areas	IQ and exposure dose^h^ Urinary fluoride and exposure dose IQ and urinary fluoride	149 (6–18)	Yes	↓	The results also reveal that exposure dose has a positive correlation with dental fluorosis (*r* = 0.299, *p* < 0.01) and urinary fluoride concentration (*r *= 0.513, *p* < 0.01) and a negative correlation with IQ (*r* = − 0.343, *p *< 0.01) along with BMI (*r* = 0.083, non-significant)
Mondal et al. (2016)	India (West Bengal)	CS	E 0.33–18.08; groundwater samples pre-monsoon and post-monsoon from borewells	IQ and water fluoride Urinary/serum fluoride and water fluoride	40 (10–14) (subpopulation of 235)	No	↓	IQ test also signifies that fluoride has a bearing on the intelligence development of the study area school children
Sharma et al. (2016)^i^	India (District Una, Himachal Pradesh)	CS^a^	E 0.40–0.68; water sample from selected schools	Fluorosis level and water fluoride IQ and fluorosis level	270 (10–14)	No	↓	As the fluorosis level increased, the proportion of children with lower intelligence increased. Based on the findings, the chronic exposure to high levels of fluoride can be one of the factors that influence intellectual development
Bashash et al. (2017)	Mexico (Mexico City)	L	E^k^	Child´s cognitive function^l^ and MUF^m^	287/211 (4/6–12)	Yes	↓	Higher prenatal fluoride exposure, in the general range of exposures reported for other general population samples of pregnant women and non-pregnant adults, was associated with lower scores on tests of cognitive function in the offspring at age 4 and 6–12 years
Razdan et al. (2017)^n^	India (Mathura district)	CS	E 0.60 vs. 1.70 vs. 4.99; water samples from different villages surrounding Mathura District from the hand pumps noted to be the source of consumed water for the inhabitant	IQ and water fluoride	219 (12–14)	No	↓	Concentration of fluoride in the ingested water was significantly associated with the IQ of children. It has also coined the proportional variability in mental output in accordance to the ingested fluoride level
Mustafa et al. (2018)	Sudan (Khartoum State)	CS^a^	E 0.14–2.07 (dry season), 0.01–1.34 (rainy season), 0.08–1.17 (average of seasons); groundwater samples (dry and rainy season) from different rural areas in Khartoum state	Schooling performance^o^ and water fluoride	775 (6–14)	No	↓	There may be an inverse relationship between fluoride levels in drinking water and schooling performance
Yu et al. (2018)	China (Tianjin)	CS	E M: 0.50 (± 0.27) vs. 2.00 (± 0.75); water samples from the public water supplies in each village	IQ and water fluoride Urinary fluoride and water fluoride	2886 (7–13)	Yes	↓	The study suggests threshold and saturation effects of moderately excessive fluoride exposure on dental fluorosis and intelligence loss in children, and a potential association between dental fluorosis and the loss of excellent intelligence
Sharma et al. (2018c)	India (District Una, Himachal Pradesh)	CS	E 0.31–0.68; water samples were collected from the selected schools	IQ and water fluoride	600 (10–14)	No	→	Risk of dental caries and DAI^p^ were more prevalent in areas with high fluoride level in water. Low intelligence level of adolescents was not significantly associated with high fluoride level hence indicating towards multifactorial causation of disease
Naik et al. (2018)	India (Mysore district)	CS	E < 1.2 vs. 1.2–2 vs. >2; water samples from different villages surrounding Mysore District from the hand pumps noted to be the source of consumed water for the inhabitant	IQ and water fluoride	264 (12–15)	No	↓	Fluoride concentration in drinking water was negatively correlated with IQ level of school children
Green et al. (2019)	Canada (Vancouver, Montreal, Kingston, Toronto, Hamilton, Halifax)	PC	CWF vs. E fluoride intake level in mg/day, M: 0.30 vs. 0.93; water fluoride concentration in mg/L, M: 0.13 vs. 0.59; data measured automatically in the water treatment plant zone matched with participants´ postal code	IQ and maternal urinary fluoride IQ and self-reported maternal daily fluoride intake from water and beverages Maternal urinary fluoride and water fluoride/fluoride intake	512^q^ 400^r^ (3–4)	Yes	↓	Maternal exposure to higher levels of fluoride during pregnancy was associated with lower IQ scores in children aged 3 to 4 years

Search criteria were: PubMed database for epidemiological studies published between January 2012 and August 2019 with the key words in the title/abstract including ‘fluoride’ and ‘IQ’ or ‘intelligence quotient’. In addition to the PubMed search, the reference lists of included studies were checked for further trials. Publications where the full text was not available in English or not published in a journal (master thesis, dissertations etc.) were excluded (see Online Resource 2)

*BMI* body mass index, *CS* cross-sectional, *CWF* community water fluoridation: water fluoridation in areas where water is artificially fluoridated with a precise dose of fluoride as a public health prevention measure, *E* endemic fluoride occurrence originating naturally in drinking water, *(G)M* (geometric) mean, *IQ* intelligence quotient, *L* longitudinal study, *MUF* maternal urinary fluoride, *r(*^*s*^*)* (partial) correlation coefficient, *PC* prospective cohort, ↓ increasing fluoride exposure adversely affected human intelligence, →  no association between fluoride exposure and human intelligence

^a^Since in this observational study data obtained at one specific point in time was analyzed, this study was categorized as a cross-sectional study, although this was not specifically designated by the authors

^b^No specific fluoride concentration was given in the publication. The authors stated that data of children from endemic fluorosis areas were compared to that of non-fluorosis areas. Children aged 8–12 who lived in coal-burning endemic fluorosis area in Bijie City of Guizhou Province were selected and divided into two groups according to the duration of comprehensive treatments given: long-term treatment group (Xiaba Village and Qianxixiang Zhongtun Village, furnace stove was changed and comprehensive control measure of health education was carried out for more than 3 years) and short-term treatment group (Chadi Village and Maoliping Village, stoves were improved and health education time < 1 year). The children who lived in a non-fluorosis area were selected as controls in 2012

^c^The authors did not state on what basis they initially divided into “endemic fluorosis areas” and “non-fluorosis areas” (for controls), but the dental fluorosis examination of study participants revealed that the incidence rate for dental fluorosis was 0/104 in the “non-fluorosis areas” group and 505/637 in the “endemic fluorosis areas” group

^d^This cohort study revealed a 38-year follow-up of participants

^e^The IQ scores of the study participants were assessed in childhood (992 participants) and adulthood (942 participants). The childhood IQ for each study member was assessed at ages 7, 9, 11, and 13 years by means of the conducted IQ test. The IQs determined at these four ages were averaged into one measure and standardized. Adult IQ was individually assessed at age 38 years by means of the conducted IQ test

^f^Although water samples were taken from the high (Najafgarh) and low fluoride area (Defence Colony), the results were not given in the publication. The authors stated regarding the fluoride levels that there are various high fluoride areas in Delhi which include Palam village (1.2–32.5 mg/L), Nangloi (1.7–13.6 mg/L), Sagarpur (3.4–24.6 mg/L) and Najafgarh, where except for the control part the whole block is polluted

^g^Although water samples were taken, ranges of fluoride concentrations instead of specific results of the analysis were given in the publication

^h^The fluoride exposure dose was calculated by fluoride concentration (mg/L) × amount water intake per day (L/day)/body weight (kg)

^i^The authors stated that this is an interim analysis of an ongoing project

^j^Notably, this interim study statistically examined exclusively a relationship between fluorosis level and water fluoride and IQ and fluorosis level. However, the authors drew conclusions regarding the relationship between fluoride exposure and human intelligence

^k^No specific fluoride concentration was given. The authors stated that by virtue of living in Mexico, individuals participating in the study have been exposed to fluoridated salt (at 250 mg/L) and to varying degrees of naturally occurring fluoride in drinking water. Previous reports, based on samples taken from different urban and rural areas, indicate that natural water fluoride levels in Mexico City may range from 0.15 to 1.38 mg/L. Mean fluoride content for Mexico City’s water supply is not available, because fluoride is not reported as part of water quality control programs in Mexico

^l^Child intelligence was measured by the General Cognitive Index (GCI) of the McCarthy Scales of Children’s Abilities at age 4 years and full scale intelligence quotient (IQ) from the Wechsler Abbreviated Scale of Intelligence (WASI) at age 6–12 years

^m^In this study, child´s cognitive function is compared to each’s woman average of all her available creatinine-adjusted urinary fluoride concentrations during pregnancy (MUC_cr_). Creatinine-adjusted urinary fluoride concentrations were obtained for each maternally derived sample by dividing the fluoride concentration (MUF) in the sample by the sample’s creatinine concentration (MUC), and multiplying by the average creatinine concentration of samples available at each trimester (MUC_average_) using the formula: (MUF/MUC) × MUC_average_. For each woman, an average of all her available creatinine-adjusted urinary fluoride concentrations during pregnancy was computed and used as the exposure measure (MUF_cr_)

^n^The authors stated that this is a pilot study that included 10% of the total sample and was utilized to check for the feasibility of the study. However, a follow-up study that refers to this pilot study could not be identified

^o^The schooling performances were measured as the average score (%) [(100 × average mark)/total mark] and the high score prevalence (%) [(100 × no. of students scoring > 70%)/total no. of students] for each of eight subjects (Islamic studies I and II, Arabic, English, mathematics, sciences, history, and technology) and the overall score

^p^The abbreviation DAI is not specified in the publication

^q^Measurement of maternal urinary fluoride levels

^r^Self-reported maternal daily fluoride intake from water and beverage consumption

### Limitations of epidemiological studies

So far, almost all studies investigating the effect of fluoride intake on intelligence were performed in relatively poor, rural communities, e.g., in China, Iran, and Mongolia, where drinking water may contain comparatively high levels of fluoride (‘exposed population’), whereas the ‘reference populations’ often had access to water that was fluoridated at the recommended level (critically discussed by (Feldman 2014; Gelinas and Allukian 2014)). This constellation may lead to a confounding effect; rural regions with unusually high or unusually low fluoride in drinking water may be associated with a less developed health-care system, as well as lower educational and socioeconomic status. Furthermore, in these regions the overall nutritional status and the intake of essential nutrients may be lower and the exposure to environmental contaminants such as lead, cadmium, mercury, or manganese may be higher—factors that are also discussed to have a potential impact on intelligence (e.g., Carrington et al. 2019; Bouchard et al. 2011; Hibbeln et al. 2007). Conversely, relatively rich communities with access to better education and/or higher socioeconomic status may more likely invest in having high-quality drinking water, e.g., to avoid fluoride concentrations above 1.5 mg/L to decrease the risk of dental fluorosis, and can afford reduction of high fluoride concentrations through filtration. In addition, particularly low fluoride concentrations in drinking water can be rectified by fluoridation at adequate levels. However, both measures require a relatively advanced public health-care system.

Notably, only two studies published since 2012 investigated the effect of fluoride exposure in drinking water resulting from community water fluoridation (CWF) (Broadbent et al. 2015; Green et al. 2019), i.e., in areas where water is fluoridated with a precise dose of fluoride as a public health prevention measure. In contrast, most of the studies (21 of 23 studies) investigated the effect of fluoride exposure in drinking water resulting from endemically occurring fluoride. In these studies, fluoride is naturally present at varying concentrations, with minimum levels of 0.08 mg/L (Mustafa et al. 2018) and maximum levels of 18.08 mg/L (Mondal et al. 2016). To maintain electroneutrality, (drinking) water with higher concentrations of endemically occurring fluoride must contain higher concentrations of positive ions to balance out the fluoride. This may affect the pH of the water or result in greater contamination by electropositive water contaminants, for example aluminum, zinc, arsenic, lead, mercury, and other metals and metalloids. Thus, in studies of naturally occurring fluoride, it is important to control for these contaminants. On the other hand, in studies of community water fluoridation, the negative fluoride ions are balanced out in the water treatment process; therefore, other substances are unlikely to be a source of confounding.

As a measure of exposure, some studies did not use individual level exposure, i.e., by individual drinking water samples, urinary fluoride samples, or dental fluorosis measurements. Frequently, the fluoride content in drinking water in the residential area of the study participants was used as a proxy which is considered to be a notable study limitation. Furthermore, it should be noted that many studies have used creatinine-adjusted urinary fluoride concentrations to account for urinary dilution which may cause an additional bias. Findings of a systematic review and meta-analysis suggest that kidney function may be associated with IQ, and children with chronic kidney disease may have below average neurocognitive and academic outcomes (Chen et al. 2018b). An additional limitation is the often-used cross-sectional study design, which is appropriate when an acute event (e.g., asthma) and the possible source of exposure (e.g., airborne pollen) occur very close to each other, since both parameters are measured simultaneously. However, cross-sectional and ecological studies do not allow the establishment of causal relationships and are not appropriate to ultimately evaluate the effect of a chronic fluoride exposure on a parameter like human intelligence, but serve to derive hypotheses.

In contrast, prospective studies in which cohorts are followed over a period of time, and data relating to predetermined exposures and outcomes that are collected over time, are considered appropriate for inferring causality (Sutton et al. 2015).

Another aspect is the control of confounding factors, which are known to influence intelligence, by using an optimal study design, i.e., statistical adjustment. Of the 23 studies that were published since 2012, only 11 performed a statistical adjustment for potential confounding factors, and in most of these studies the included confounders were incomplete. Twelve of 23 studies aimed to consider the influence of potential confounding factors by their study design, e.g., by comparing populations with ‘similar characteristics’, did not consider the influence of confounding factors at all, or did not comment on this fact (Table 6).

### Assessment of prospective epidemiological studies

In summary, most epidemiological studies performed in rural areas reported an association of high fluoride exposure with lower intelligence. Most of these studies are of low quality (e.g., insufficient control of confounding factors, no individual level exposure assessment) and inadequately designed to prove or disprove hypotheses (cross-sectional). The results of the two available studies with a suitable study design (prospective cohort studies) conducted in non-endemic CWF areas that also appropriately considered confounding factors (even though there are still some limitations, see below) are conflicting. First of all, it has to be noted that the two studies address different questions. One study investigated the influence of fluoride exposure on the development of intelligence at various time points, ranging from infancy to adulthood (Broadbent et al. 2015). The other study examined the influence of fluoride exposure during pregnancy on the intelligence of children only once at an age between 3 and 4 years (Green et al. 2019), i.e., at an age range where performance in intelligence tests is improving quite rapidly.

The first of the two prospective cohort studies was performed with a general population sample of 1.037 children born in Dunedin, New Zealand, between 1 April 1972 and 30 March 1973 (Broadbent et al. 2015). The participants were followed for 38 years and their fluoride intake via drinking water (residence in a CWF area versus non-CWF area; 0.7–1.0 mg fluoride/L vs. 0.0–0.3 mg fluoride/L), fluoride dentifrice, and/or 0.5 mg fluoride tablets in early life (prior to age 5 years) was deduced. IQ was assessed repeatedly between ages 7 and 13 years and at age 38 years. It was reported that no statistically significant differences in IQ due to fluoride exposure were observed also following adjustment for potential confounding variables, including sex, socioeconomic status, breastfeeding, and birth weight (as well as educational attainment for adult IQ outcomes).

The second prospective cohort study conducted in Canada was performed with children born between 2008 and 2012 (Green et al. 2019). Forty-one percent lived in communities supplied with fluoridated municipal water. Samples were taken from 601 mother-child pairs and the children were between ages 3 and 4 years at intelligence testing. Maternal urinary fluoride (MUF), adjusted for specific gravity and averaged across three trimesters, was measured for 512 pregnant women, self-reported maternal daily fluoride intake from water and beverage consumption was available for 400 pregnant women. The authors concluded that maternal exposure to higher levels of fluoride during pregnancy was associated with lower full-scale IQ scores in children (Green et al. 2019). This effect was significant, albeit rather small and restricted to boys. Thus, an increase of 1 mg/L of MUF was significantly associated with a 4.49 (95% CI − 8.38 to − 0.60) lower FSIQ score in boys, whereas girls showed a slight but not significant increase in IQ scores (*B* = 2.40; 95% CI − 2.53 to 7.33). A 1-mg higher daily intake of fluoride among pregnant women was significantly associated with a 3.66 lower IQ score (95% CI − 7.16 to − 0.14) in boys and girls. However, it should be mentioned that mean FSIQ was the same among children from non-fluoridated (108.07) and fluoridated (108.21) areas. It was only after splitting the analysis by sex that the authors obtained an association among boys, for urinary fluoride.

Since the two available prospective studies led to different results (Broadbent et al. 2015; Green et al. 2019), we systematically compared features that may explain the discrepancy (Table 7). A limitation of both studies is the lack of IQ data of the mothers, because parental IQ is a strong confounder. Moreover, it cannot be excluded that the ‘outcome’ (intelligence) influenced fluoride exposure in the study of Green et al. (2019). An additional limitation of the study performed by Green et al. (2019) is that the intelligence tests have been performed only once between the age of 3 and 4 years, but the exact age of the children at the time point of the test has not been considered in the statistical analysis. This may be problematic, because the IQ of children changes strongly between 3 and 4 years. Moreover, the Wechsler Preschool and Primary Scale of Intelligence Test (WPPSI-III) used in the study provides different sets of subtests for the 2:6–3:11 (years:months) age band and the 4:0–7:7 age band. In contrast, Broadbent et al. (2015) assessed IQ at ages 7, 9, 11, and 13 years and used an average. Therefore, this study evaluated intelligence at older age compared to Green et al. (2019), but obtained a more robust measure of intelligence. Broadbent et al. (2015) used a complete birth cohort with 91% of eligible births, representing a very high rate. In contrast, only 610 of 2001 pregnant women from the MIREC program were considered in Green et al. (2019); moreover, information on maternal urinary fluoride was missing in a relatively high fraction of the mothers of children of whom IQ was determined. This may represent a possible source of bias. Furthermore, this study used creatinine-adjusted urinary fluoride concentrations to account for urinary dilution which may cause an additional bias if a study participant suffered from renal problems influencing the IQ (Chen et al. 2018b). Broadbent et al. (2015) studied the influence of possible confounding factors and obtained significant associations of socioeconomic status, breastfeeding, and low birth weight with the IQ. These factors were used to adjust the analysis of community water fluoridation with IQ (Broadbent et al. 2015). As indicated by the authors (Broadbent et al. 2015), a limitation of the study is the fact that individual water-intake level was not directly measured and dietary fluoride was not considered. Green et al. (2019) did not consider breastfeeding and low birth weight as possible confounders (both factors significantly associated with IQ in the study of Broadbent); they considered some of the relevant confounders (city, socioeconomic status, maternal education, race/ethnicity, prenatal secondhand smoke exposure), but did not adjust for others (alcohol consumption and further dietary factors, other sources of fluoride exposure, exact age of children at time point of testing). Furthermore, the study (Green et al. 2019) did not include assessment of children’s postnatal fluoride exposure via, e.g., diet, fluoride dentifrice, and/or fluoride tablets, which is considered to be a noteworthy limitation.

**Table 7:** Comparison of prospective epidemiological studies

Broadbent et al. (2015)	Green et al. (2019)
*Study design*	
Prospective study Children in areas of residence with and without community water fluoridation (CWF) at age of 3 and/or 5 years General population-based study of children born in Dunedin, New Zealand Complete birth cohort of consecutive births between April 1, 1972–March 31, 1973 with 1037 children (91% of eligible births) and 95.4% retention after 38 years of prospective follow-up Assessment of IQ at ages 7, 9, 11 and 13 years by the Wechsler Adult Intelligence Scale-Revised (averaged into 1 measure) Assessment of IQ at 38 years by the Wechsler Adult Intelligence Scale-Revised test	Prospective study *N* = 2001 pregnant women were recruited in the MIREC program from 10 cities in Canada between 2008 and 2011. From the children a subset of *n* = 610 was selected because of “budgetary restraints” Maternal urinary fluoride was measured in urine spot samples at each trimester and a mean was obtained IQ was available in 601 children analyzed between age 3 to 4 years by the Wechsler Primary and Preschool Scale of Intelligence-III Finally, maternal urinary fluoride concentrations and IQ data of the children were available for 512 mother-child pairs
*Main result*	
Fluoride concentrations in drinking water of areas with CWF ranged between 0.7 and 1.0 mg/L (ppm); without CWF between 0.0–0.3 mg/L. Community water fluoridation was not associated with intelligence; the statistical analysis was adjusted for: sex, socioeconomic status, low birth weight, and breastfeeding; analysis of adult IQ also adjusted for educational achievements	Urinary fluoride concentrations of the mothers were 0.51 (0.33–0.62) mg/L (mean; 25th–75th percentile range). An increase of 1 mg/L maternal urinary fluoride was associated with a 4.49 lower IQ score in boys (*p* = 0.02), but not in girls (*p* = 0.33). This analysis was adjusted for city, HOME score, maternal education, ethnicity, child sex and prenatal secondhand smoke exposure
*Further factors analyzed for an association with IQ*	
Sex: n.s Socioeconomic status: *p* < 0.001 Breastfeeding (in areas with CWF): *p* < 0.001 Low birth weight: *p* < 0.024 Educational attainment (for adult IQ): *p* < 0.001 Fluoride toothpaste: n.s Fluoride tablets: n.s	*P* < 0.001 (girls had 4.95 higher IQ scores than boys) Not reported Not reported Not reported Not reported Not reported Not reported

*n.s.* not significant

Green et al. (2019) present the intelligence of individual children in scatter plots showing maternal urinary fluoride concentration versus IQ (Fig. 3A in Green et al. 2019). Here, male children show a decrease in IQ with increasing maternal urinary fluoride concentration, while female children show a non-significant increase. It should also be noted that the influence of fluoride (increase from the 10th to the 90th percentile of maternal urinary fluoride concentration) of 3.14 IQ in boys is relatively small, compared to the mean difference of around 5 between boys and girls (Green et al. 2019).

Considering the limitations of so far available epidemiological studies, it is difficult to adequately interpret their findings since they present heterogeneous results with a high risk of bias. The only two studies with an appropriate study design (Broadbent et al. 2015; Green et al. 2019) differed in important characteristics (Table 7). The available epidemiological evidence does not provide sufficient arguments to raise concerns with regard to CWF in the range of 0.7–1.0 mg/L, and to justify the conclusion that fluoride is a human developmental neurotoxicant that should be categorized as similarly problematic as lead or methylmercury at current exposure levels.

For final clarification, prospective studies of even higher quality would be required. All previously reported confounders should be considered (Table 8), including the confounders analyzed in the study of Broadbent et al (2015), namely socioeconomic status, breastfeeding, low birth weight, educational attainment, fluoride toothpaste, and fluoride medication. Furthermore, also the influence of the IQ of the mothers should be analyzed in the future. The association of the confounders with IQ should be presented separately for each confounder, and (at least) all influential confounders should be included into the multivariate analysis. The exact ages of the children should be considered, particularly when IQ tests are performed at young age.

**Table 8:** Examples of confounding factors that should be considered in epidemiological studies on a possible association between fluoride exposure and intelligence

Residence, particularly urban versus rural areas
Water improvement plants: is fluoride, lead, or arsenic removed from drinking water?
Breastfeeding; breastfed children may have higher IQs
Confounding by other sources of fluoride (e.g., dental products) than drinking water
Background of parents: educational level, socioeconomic status, income, IQ
Birth weight; first-born?
Intake of iodine
Exposure to other chemicals: lead, methylmercury, arsenic, polychlorinated biphenyls

### Assessments by other bodies

The effect of fluoride on human intelligence has already been assessed by different governmental organizations. For example, in 2006 the NRC evaluated epidemiologic studies of populations exposed to different concentrations of fluoride, as well as individual case studies (NRC 2006). According to NRCs evaluation, results of available studies, all performed in China, are not considered relevant for the US population, since most of the publications were brief reports and omitted important study details (e.g., modifications of a standard IQ test were not specified). Nevertheless, due to studies reporting that the average IQ scores were lower in more highly exposed children, the NRC report indicated that additional research is warranted to determine the effects of fluoride on intelligence.

More recently, the Health Research Board of Ireland conducted a systematic review which includes IQ and neurological manifestations (Sutton et al. 2015). The assessment differentiated between fluoride non-endemic areas or areas with CWF and fluoride-endemic areas. The above already discussed prospective cohort study (Broadbent et al. 2015) was identified and considered to have an adequate experimental design (Sutton et al. 2015). For fluoride-endemic areas, a summary of the available studies suggests that children living in areas with naturally occurring high fluoride in the water (higher than the CWF levels of 0.4–1 mg/L) have a lower IQ compared to children drinking water with naturally occurring levels of fluoridation, which are similar to the CWF levels in Ireland (Sutton et al. 2015). However, the authors stated that the quality of the studies was poor and the study design inadequate to prove or disprove a causal relationship which is in line with the present evaluation.

The National Health and Medical Research Council (NHMRC) of the Australian Government reviewed the epidemiological evidence until 2016. According to the NHMRC Public Statement 2017, there is reliable evidence that community water fluoridation at current Australian levels of 0.6–1.1 mg/L is not associated with cancer, Down syndrome, cognitive dysfunction, lowered intelligence or hip fracture (NHMRC 2017b).

Furthermore, a retrospective cohort study was conducted by the Institute for Evaluation of Labour Market and Education Policy (IFAU), which is a research institute under the Swedish Ministry of Employment (Aggeborn and Oehman 2017). In this study, the effects of fluoride exposure by drinking water throughout life on cognitive and non-cognitive ability, math test scores, and labor market outcomes were investigated in a nationwide large-scale setting. The Swedish register dataset for the cohorts born 1985–1992 was used, together with drinking water fluoride data. Water fluoride concentrations were estimated utilizing the geographic location of the current residence and this was linked to water supply with known fluoride content. The fluoride data are based on exposure from drinking water which had fluoride levels ranging from effectively 0 up to 1.5 mg/L. Cognitive development was measured through the results from the national math test taken at around the age of 16 years in the ninth grade. Further cognitive and non-cognitive ability measures originated from the Swedish military enlistment (Aggeborn and Oehman 2017). The authors reported that they estimated zero effects on cognitive ability, non-cognitive ability, and math test scores for fluoride levels in Swedish drinking water (Aggeborn and Oehman 2017).

## In vitro studies

In vitro studies are usually performed to examine the mechanisms of action of a test compound. In the present review, we analyzed concentration ranges in which fluoride caused cytotoxic effects or influenced other end points, e.g., gene expression. In an explorative approach, these concentrations were compared with human plasma concentrations of fluoride. Importantly, this approach can only be considered as an approximate estimation, because cells cultured in vitro do not necessarily show the same susceptibility as cells organized in tissues in vivo.

We searched the PubMed database with key words, including fluoride and/or NaF in different combinations with toxic, toxicity, neurotoxic, neurotoxicity, cells, in vitro, neuro-2 A cells, embryonic stem cells, ESC, hESC, neural progenitor cells, NPC, hNPC, neural stem cells, pluripotent stem cells, PSC, hiPSC, primary hippocampal neurons, pheochromocytoma cells, PC 12 cells, BV-2 microglial cells, astrocytes, human neuroblastoma SH-SY5Y cells or human neuroblastoma SK-N-SH cells, neural crest cells, and NCC. In total, 26 in vitro studies on fluoride were found (Table 9).

**Table 9:** Fluoride concentrations of in vitro studies that caused positive results in neuronal cells and precursor/stem cells

Cell type	Fluoride concentrations		Endpoint	References
	Highest concentration without effect	Lowest concentration with effect		
Neuro-2 A cells	2 mM	4 mM	Altered expression of neuronal genes	Chen et al. (2017a)
Human embryonic stem cells (H9)	0.5 mM	1 mM	Altered gene expression	Fu et al. (2016)
		2 mM	Cytotoxicity	
Mouse embryonic stem cells (D3)		1 mM	Cytotoxicity	Nguyen Ngoc et al. (2012)
Primary hippocampal neurons of rats	0.48 mM	0.95 mM	Cytotoxicity	Zhang et al. (2007)
Primary hippocampal neurons of rats		0.48 mM	Olive tail moments elevated Up-regulation of NF-kappaB (*p* < 0.05) DNA damage	Zhang et al. (2008)
		0.95 mM	Increase of DNA in the tail Up-regulation of NF-kappaB (*p* > 0.05) S-phase cell-cycle arrest	
Primary hippocampal neurons of rats		0.48 mM	NCAM-140 expression level decreased	Xia et al. (2007)
		0.95 mM	NCAM expression level decreased	
		1.91 mM	NCAM-120 expression level decreased Cell survivor decreased	
Primary hippocampal neurons of mouse		0.12 mM	Intracellular Ca^2+^ fluxes > increase of intercellular concentration Increase of apoptotic peaks	Haojun et al. (2012)
PC12 cells (pheochromocytoma cells)		0.5 mM	Cytotoxicity (8 h)	Ke et al. (2016)
		1 mM	Cytotoxicity (2 h) Increased expression levels of apoptosis-related proteins (p-elF, PARP) Increased expression levels of ERS-related protein XBP-A	
PC 12 cells (pheochromocytoma cells)		0.005 mM	Intracellular ROS increase Apoptotic cells Cytotoxicity (48 h)	Zhang et al. (2015b)
		0.05 mM	Apoptotic cells Cytotoxicity (8 h)	
		0.5 mM	Apoptotic cells Cytotoxicity (2 h)	
PC 12 cells (pheochromocytoma cells)	0.024 mM	0.24 mM	Decline of MTT reduction Protein oxidation	Shan et al. (2004)
PC 12 cells (pheochromocytoma cells)	0.024 mM	0.24 mM	Selective decreases in the number of nAChRs	Chen et al. (2003)
PC 12 cells (pheochromocytoma cells)	0.024 mM	0.24 mM	Increase of total inositol phosphates	Bencherif and Lukas (1991)
BV-2 microglia cells	0.5 mM	1 mM	Increase of IL-6 concentration	Chen et al. (2017b)
		2 mM	Significant decrease of cell viability (12 h) SOD activity lower Increase of TNF-α level	
BV-2 microglia cells	0.024 mM	0.119 mM	Increased JNK phosphorylation level	Yan et al. (2013)
		1.19 mM	Cytotoxicity Increase of NO release Increase of TNFα release	
BV-2 microglia cells		0.024 mM	SOD activities decreased NOS (synthesizing NO) increased	Shuhua et al. (2012)
		0.119 mM	Change into activated microglia Up-regulated OX-42 expression	
		1.19 mM	Cytotoxicity	
Bergmann glia cells	0.5 mM	1 mM	Cytotoxicity	Flores-Mendez et al. (2014)
Hippocampal slices of rat and mouse		1 to about 5 mM	Activation of MAP kinase Followed by volume reduction	Lee et al. (2016)
Astrocytes of rat cerebral cortex		1 mM	Cell cycle arrest transited from S phase to G2/M phase Increase of subG1 cells	Li et al. (2010)
Human neuroblastoma SH-SY5 Y cells		0.48 mM	Drp1 expression increased Fis 1 expression reduced Mfn1 protein increased Mfn2 protein increased	Zhao et al. (2019)
		0.95 mM	Drp1 expression reduced Fis 1 expression reduced Mfn1 expression increased Mfn2 expression increased	
Human neuroblastoma SH-SY5 Y cells	0.71 mM	0.95 mM	Cytotoxicity Induced apoptosis	Tu et al. (2018)
Human neuroblastoma SH-SY5 Y cells	0.48 mM	0.95 mM	SYN expression reduced TrkB expression reduced p-Erk expression increased	Chen et al. (2018a, b)
		1.43 mM	PSD 95 protein expression reduced BDNF protein expression increased	
Human neuroblastoma SH-SY5 Y cells	0.48 mM	0.95 mM	Autophagic vesicles decreased	Tang et al. (2017)
Human neuroblastoma SH-SY5 Y cells	0.48 mM	0.95 mM	LDH levels higher	Xu et al. (2013)
Human neuroblastoma SH-SY5 Y cells	0.48 mM	0.95 mM	Cytotoxicity Percentages of apoptosis higher Activity of caspase-3 higher mRNA expression levels for Fas, Fas-L, and caspases (3 and 8) higher	Xu et al. (2011)
Human neuroblastoma SH-SY5 Y cells		0.12 mM	Cytotoxicity (24 h) Expression of phospho-JNK	Liu et al. (2011)
		1.91 mM	Cytotoxicity (8 h) MTT reduction	
Human neuroblastoma SK-N-SH cells		~ 2.5 mM	Stimulation of inositol phosphates release	Fisher et al. (1993)

Cytotoxicity was observed at concentrations of approximately 1 mM in most studies. However, a few studies reported cytotoxic effects at concentrations as low as 0.12 mM, such as apoptosis reported in primary hippocampal neurons of mice (Haojun et al. 2012) or in human neuroblastoma cells (Liu et al. 2011); (Table 9). One exception is a more recent study (Zhang et al. 2015b) that reported reduced viability of PC12 cells at the extremely low concentration of 0.005 mM which would be lower than the concentration in drinking water (up to 1.5 mg/L corresponding to 0.079 mM). However, the effects of 0.005 mM fluoride on cell viability are inconsistent with regard to incubation period. After 8 h of fluoride treatment, cell proliferation was slightly decreased, after 12–24 h cells appeared to recover and cell survival rate was higher than that of the control groups, and after 48 h cell survival rates declined again. This questions the proposed relationship between lower survival rate and fluoride concentration observed after 48 h.

Positive results for readouts other than cytotoxicity, such as gene expression, up-regulation of the inflammatory factor NF-kappaB, or altered intracellular Ca^2+^ flux, were typically observed between 0.12 and 4 mM depending on the cell system and end point. In contrast, decreased superoxide dismutase (SOD) and increased nitric oxide synthase (NOS) activities were observed in BV-2 microglia cells at the low concentration of 0.024 mM, which appears to be an exception and must be interpreted with caution unless reproduced by independent studies (Shuhua et al. 2012).

In summary, the effects on cell systems including cytotoxicity, gene expression, and further readouts were typically obtained at concentrations ranging between 0.1 and 4 mM, i.e., orders of magnitude higher than plasma fluoride levels detected in humans.

## Conclusions

For risk evaluation, we compared human exposure (expressed as mg fluoride/kg b.w./day) and no observed adverse effect levels (NOAELs) derived from animal experiments (also as mg fluoride/kg b.w./day). The adequate daily fluoride intake (AI) is 50 µg/kg b.w./day (EFSA 2013), and in the EU, the median fluoride intake from water has been estimated to be 1.86 µg/kg b.w./day, with reports of rare extreme levels of 120 µg/kg b.w./day (EFSA 2013) (Fig. 1). This extreme scenario (120 µg/kg b.w./day) corresponds to a 70 kg person drinking 2 L with 4.2 mg/L fluoride (or slightly lower concentrations if one considers the additional contribution by food and dental care products). The average intake of fluoride from food in European countries is approximately 5–28 µg/kg b.w./day and toothpaste may contribute approximately 1.4 µg/kg b.w./day in adults and 11.5 µg/kg b.w./day in children (EFSA 2013). Therefore, it seems pragmatic to use the recommended daily intake of 50 µg/kg b.w./day to compare NOAELs from animal experiments, while also considering the extreme scenario with 120 µg/kg b.w./day. The lowest reported NOAEL from a well-designed chronic animal toxicity study investigating systemic effects was 2.5 mg/kg b.w./day fluoride (Fig. 2), resulting in a margin of exposure (MoE) of 50 compared to the adequate daily intake (50 µg/kg b.w./day). For the extreme scenario of 120 µg/kg b.w./day, the MoE would be 21. With the NOAEL of 8.5 mg/kg b.w./day as point of departure for developmental toxicity, the adequate daily intake of 50 µg/kg b.w./day resulted in a high MoE of 170 (Fig. 2). Due to serious study limitations, the lower LOAELs/NOAELs reported in some recent studies on developmental or neurobehavioral toxicity are not considered appropriate to derive an MoE, but warrant further investigations.

In addition to the above-described approach, internal fluoride exposure (plasma concentrations) was compared to concentrations that caused cytotoxicity or other test results (e.g., gene expression changes) in vitro in neuronal or precursor/stem cells (Table 9). Most in vitro studies resulted in measurable results at approximately 1 mM fluoride with a range of approximately 0.1–4 mM, which is 333-fold higher than the highest fluoride concentration of 3 µM reported in healthy adults. For individuals consuming drinking water with extremely high fluoride concentrations (> 8 mg fluoride/L), plasma concentrations of approximately 10 µM F^−^ have been reported (Fig. 1) (Jha et al. 1982). This is still 100-fold below the critical in vitro cytotoxic concentration of 1 mM fluoride. The particularly low concentration of 0.1 mM fluoride reported in some neuronal cells to induce apoptotic effects in vitro (Haojun et al. 2012; Liu et al. 2011) would yield an ‘in vitro MoE’ of 33 compared to the plasma fluoride concentration of 3 µM, still more than one order of magnitude higher than plasma fluoride concentrations found in healthy humans. Furthermore, fluoride concentrations in human brain tissue have been reported to be lower than those reported in plasma (Taves et al. 1983). It also should be taken into consideration that the in vitro data presented here were obtained in cultivated neuronal or precursor cells, which may show different susceptibilities compared to tissues in vivo. Nevertheless, this in vitro approach allows a first assessment of the order of magnitude where adverse effects may be expected. It thus supports the NOAEL based risk evaluation described above.

This review considered experimental in vitro and animal studies as well as epidemiological studies. Of note, the majority of epidemiological studies reported an association between lower measures of intelligence and high fluoride exposure. However, the experimental evidence suggests that current exposure to fluoride, even for individuals with relatively high fluoride intake, is clearly below levels that lead to adverse effects in vitro or in animals. The discrepancy between experimental and epidemiological evidence may be reconciled with deficiencies inherent in most epidemiological studies on a putative association between fluoride and intelligence, especially with respect to adequate consideration of potential confounders. The only two prospective cohort studies conducted in areas with community water fluoridation that considered possible confounding factors reported conflicting results (Broadbent et al. 2015; Green et al. 2019). Overall, despite the remaining uncertainties, and based on the totality of evidence the present review does not support the presumption that fluoride should be considered as a human developmental neurotoxicant at current exposure levels in European countries.

## Research needs

For a comprehensive risk assessment, further research is needed. Human exposure to fluoride has already been studied in the past (EFSA 2013; FSAI 2018), but to enable a more accurate assessments of total fluoride intake and of fluoride intake from different sources it is recommended to systematically analyze the fluoride content of foods, beverages, and water for human consumption in the EU using a standardized methodology. Furthermore, the validation of biomarkers of actual and chronic fluoride intake could contribute to an overall exposure assessment. In recent years, several developmental and neurobehavioral animal studies reported unusually low NOAELs and LOAELs. However, a critical analysis of these studies showed that they often did not comply with state-of-the-art scientific quality criteria. For clarification, sufficiently powered high-quality animal studies would be helpful. Similarly, high-quality prospective epidemiological studies are required that adequately control for any confounding factors.

## Electronic supplementary material

Below is the link to the electronic supplementary material.

Supplementary file1 (PDF 750 kb)

Supplementary file2 (PDF 13 kb)
