# Supplementary material for: Toxicity of fluoride: critical evaluation of evidence for human developmental neurotoxicity in epidemiological studies, animal experiments and in vitro analyses
Source: Arch Toxicol. 2020 May 8;94(5):1375–415. doi: 10.1007/s00204-020-02725-2 (PMC7261729; doi:10.1007/s00204-020-02725-2)
Supplement: Supplementary file 1 — Supplementary file1 (PDF 750 kb) [file 204_2020_2725_MOESM1_ESM.pdf]

Online Resource 1

Archives of Toxicology

Toxicity of fluoride: critical evaluation of evidence for human developmental neurotoxicity in epidemiological studies, animal experiments and in vitro analyses

Sabine Guth, Stephanie Hüser, Angelika Roth, Gisela Degen, Patrick Diel, Karolina Edlund, Gerhard Eisenbrand, Karl-Heinz Engel, Bernd Epe, Tilman Grune, Volker Heinz, Thomas Henle, Hans-Ulrich Humpf, Henry Jäger, Hans-Georg Joost, Sabine E. Kulling, Alfonso Lampen, Angela Mally, Rosemarie Marchan, Doris Marko, Eva Mühle, Michael A. Nitsche, Elke Röhrdanz, Richard Stadler, Christoph van Thriel, Stefan Vieths, Rudi F. Vogel, Edmund Wascher, Carsten Watzl, Ute Nöthlings, Jan G. Hengstler

Corresponding authors:

Jan G. Hengstler: Leibniz Research Centre for Working Environment and Human Factors (IfADo), Depart. of Toxicology, Dortmund, Germany. Tel.: +49-231-1084-348. E-Mail: hengstler@ifado.de

Ute Nöthlings: Department of Nutrition and Food Sciences, Nutritional Epidemiology, Rheinische Friedrich-Wilhelms University Bonn, Bonn, Germany. Tel.: +49-0228-73-60490. E-Mail: noethlings@uni-bonn.de

Online Resource 1 Reports on possible health issues of fluoride from different media outlets obtained online.

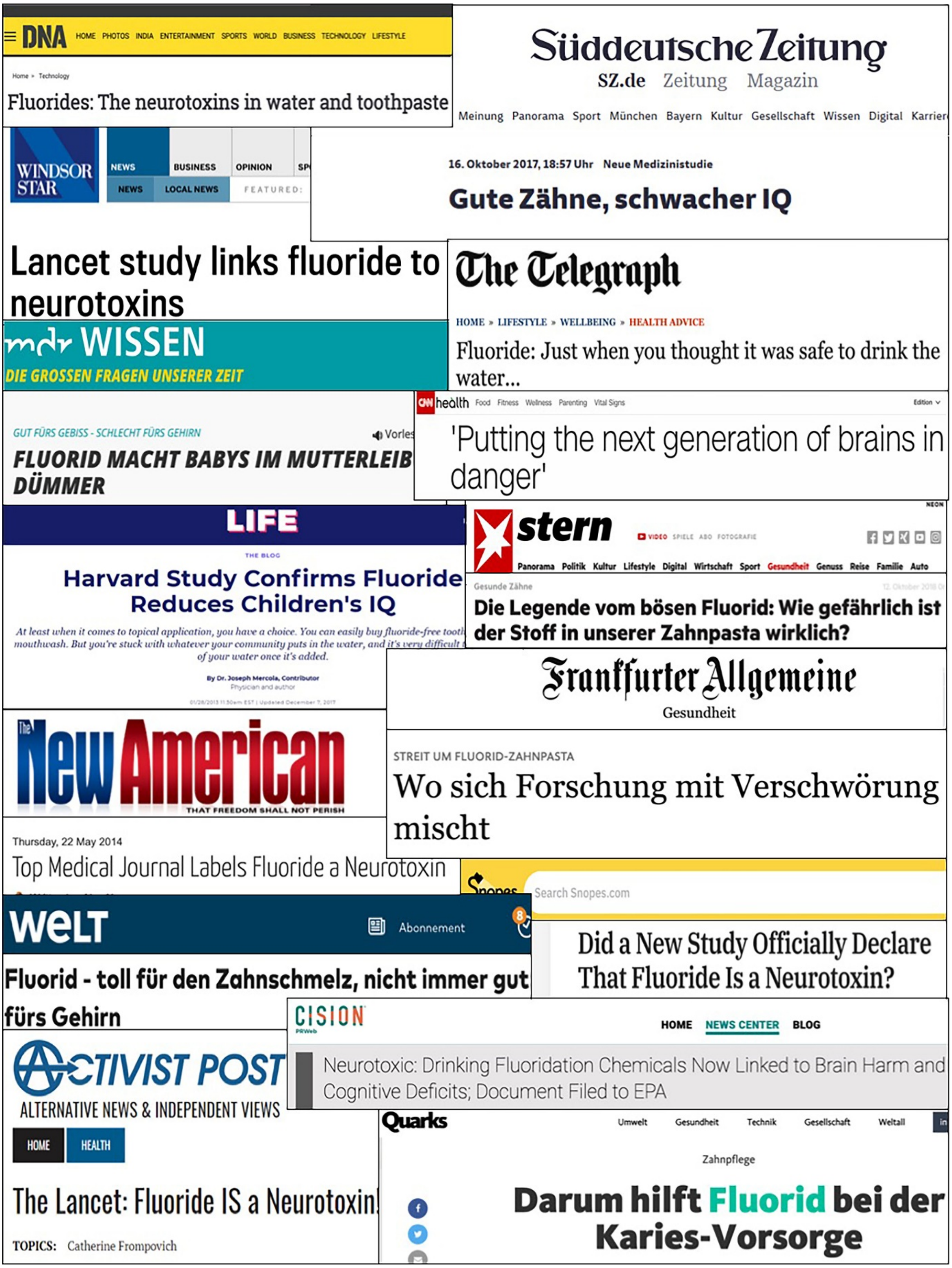

A non-representative internet review was conducted to assess how media and freely accessible online information platforms (i.e. web blogs, press agencies or industrial press releases) report on the possible neurotoxicological potential of fluoride between 2012-2018. For this purpose, different databases (“Nexis Uni”, “Genios”, also “Google Advanced Search”) were used. The following keywords were applied for news search (in English and German): “fluorid* AND neurotoxi*” as well as “fluorid* AND intelligen*” and “fluorid* AND IQ*”. The period from 01/01/2012 to 12/31/2018 was chosen for each search request. More than 2.500 hits could be identified for the Nexis Uni search “fluorid* AND intelligen*”, about 1.040 for the Nexis Uni search “fluorid* AND IQ*”. Using the keywords “fluorid* AND neurotoxi*” about 670 hits were still obtained in the Nexis Uni database. Five articles were obtained that contained “fluorid*” and “neurotox*” in their headline – two newspapers, one news magazine, one online press release service and a web blog (1,2,5,6,14). Searching in the German database provider Genios for news that contain “Fluorid* AND “Neurotoxi*” ten press articles could be found. In general, the role of fluoride in reproduction toxicology is a topic that receives much attention in the internet. On the one hand, several pages, mostly web blogs or interest-led platforms, but also few examples of mass media, claimed that fluoride is classified as a human neurotoxin or they present information in a biased way that is characterised by e.g. emotional, alarming language, misleading headlines and by a lack of presenting also contrary opinions. On the other hand, there are also some examples of news articles that present information in a more balanced way, e.g. by quoting different experts or by discussing also the limitations of studies. Additionally, also some examples of articles from leading newspapers were identified that made the often heated and not fact-driven debates about fluoride the subject of their stories (7, 8, 12).

Examples:

1. https://www.dnaindia.com/technology/report-fluorides-the-neurotoxins-in-water-and-toothpaste-1994325
2. https://windsorstar.com/news/local-news/lancet-study-links-fluoride-to-neurotoxins
3. https://www.mdr.de/wissen/mensch-alltag/fluorid-macht-kinder-dumm-100.html
4. https://www.huffpost.com/entry/fluoride_b_2479833
5. https://www.thenewamerican.com/usnews/health-care/item/18324-top-medical-journal-labels-fluoride-a-neurotoxin
6. https://www.activistpost.com/2014/06/the-lancet-fluoride-is-neurotoxin.html
7. https://www.telegraph.co.uk/lifestyle/wellbeing/healthadvice/10722701/Fluoride-Just-when-you-thought-it-was-safe-to-drink-the-water....html
8. https://www.stern.de/gesundheit/zaehne/fluorid-in-zahncreme--die-legende-vom-gift-in-der-zahnpasta-8395952.html
9. https://edition.cnn.com/2014/02/14/health/chemicals-children-brains/index.html
10. https://www.sueddeutsche.de/politik/neue-medizinistudie-gute-zaehne-schwacher-iq-1.3710529
11. https://www.snopes.com/fact-check/new-study-officially-declare-fluoride-neurotoxin/
12. https://www.faz.net/aktuell/gesellschaft/gesundheit/fluorid-in-zahnpasta-ist-es-wirklich-schaedlich-und-giftig-15454692.html
13. https://www.quarks.de/gesundheit/darum-solltest-du-es-mit-fluorid-nicht-uebertreiben/
14. https://www.prweb.com/releases/2016/tscapetition/prweb13920498.htm
15. https://www.welt.de/gesundheit/article169841727/Fluorid-toll-fuer-den-Zahnschmelz-nicht-immer-gut-fuers-Gehirn.html
