# Supplementary material for: Toxicity of fluoride: critical evaluation of evidence for human developmental neurotoxicity in epidemiological studies, animal experiments and in vitro analyses
Source: Arch Toxicol. 2020 May 8;94(5):1375–415. doi: 10.1007/s00204-020-02725-2 (PMC7261729; doi:10.1007/s00204-020-02725-2)
Supplement: Supplementary file 2 — Supplementary file2 (PDF 13 kb) [file 204_2020_2725_MOESM2_ESM.pdf]

Online Resource 2

Archives of Toxicology

Toxicity of fluoride: critical evaluation of evidence for human developmental neurotoxicity in epidemiological studies, animal experiments and in vitro analyses

Sabine Guth, Stephanie Hüser, Angelika Roth, Gisela Degen, Patrick Diel, Karolina Edlund, Gerhard Eisenbrand, Karl-Heinz Engel, Bernd Epe, Tilman Grune, Volker Heinz, Thomas Henle, Hans-Ulrich Humpf, Henry Jäger, Hans-Georg Joost, Sabine E. Kulling, Alfonso Lampen, Angela Mally, Rosemarie Marchan, Doris Marko, Eva Mühle, Michael A. Nitsche, Elke Röhrdanz, Richard Stadler, Christoph van Thriel, Stefan Vieths, Rudi F. Vogel, Edmund Wascher, Carsten Watzl, Ute Nöthlings, Jan G. Hengstler

Corresponding authors:

Jan G. Hengstler: Leibniz Research Centre for Working Environment and Human Factors (IfADo), Depart. of Toxicology, Dortmund, Germany. Tel.: +49-231-1084-348. E-Mail: hengstler@ifado.de

Ute Nöthlings: Department of Nutrition and Food Sciences, Nutritional Epidemiology, Rheinische Friedrich-Wilhelms University Bonn, Bonn, Germany. Tel.: +49-0228-73-60490. E-Mail: noethlings@uni-bonn.de

Online Resource 2 Excluded epidemiological studies

**Reference**	**Reason for exclusion**
(Bai et al. 2014)	Only abstract in English available
(Malin and Till 2015)	ADHA as endpoint
(Li et al. 2016)	Cognitive function as endpoint
(Valdez Jimenez et al. 2017)	Mental and psychomotor development as endpoint
(Barberio et al. 2017)	Learning disability as endpoint
(Bashash et al. 2018)	ADHA as endpoint

Bai A, Li Y, Fan Z, Li X, Li P (2014) Intelligence and growth development of children in coal-burning-borne arsenism and fluorosis areas: an investigation study. Chinese Journal of Endemiology 33(2):160-163 doi:10.3760/cma.j.issn.2095-4255.2014.02.011

Barberio AM, Quinonez C, Hosein FS, McLaren L (2017) Fluoride exposure and reported learning disability diagnosis among Canadian children: Implications for community water fluoridation. Can J Public Health 108(3):e229-e239 doi:10.17269/cjph.108.5951

Bashash M, Marchand M, Hu H, et al. (2018) Prenatal fluoride exposure and attention deficit hyperactivity disorder (ADHD) symptoms in children at 6-12years of age in Mexico City. Environ Int 121(Pt 1):658-666 doi:10.1016/j.envint.2018.09.017

Li M, Gao Y, Cui J, et al. (2016) Cognitive Impairment and Risk Factors in Elderly People Living in Fluorosis Areas in China. Biol Trace Elem Res 172(1):53-60 doi:10.1007/s12011-015-0568-0

Malin AJ, Till C (2015) Exposure to fluoridated water and attention deficit hyperactivity disorder prevalence among children and adolescents in the United States: an ecological association. Environ Health 14:17 doi:10.1186/s12940-015-0003-1

Valdez Jimenez L, Lopez Guzman OD, Cervantes Flores M, et al. (2017) In utero exposure to fluoride and cognitive development delay in infants. Neurotoxicology 59:65-70 doi:10.1016/j.neuro.2016.12.011
